# Evaluation of Oxford Nanopore Technologies workflows for genomic epidemiology of outbreak-associated bacterial isolates in the clinical setting

**DOI:** 10.1099/mgen.0.001626

**Published:** 2026-02-10

**Authors:** Stefan Neuenschwander, Loïc Borcard, Sonja Gempeler, Miguel A. Terrazos Miani, Carlo Casanova, Alban Ramette

**Affiliations:** 1 Institute for Infectious Diseases, University of Bern, Bern, Switzerland; 2 Multidisciplinary Center for Infectious Diseases, University of Bern, Bern, Switzerland

**Keywords:** bacterial pathogens, core-genome multi-locus sequence typing (cgMLST), clinical setting, genome assembly, genomic epidemiology, outbreak, Oxford Nanopore Technologies, plasmids, R10.4.1

## Abstract

Accurate and efficient whole-genome sequencing (WGS) is crucial for clinical diagnostics and surveillance of bacterial infections. Here, we investigate the potential of a new Oxford Nanopore Technologies (ONT) workflow for WGS of clinically relevant bacterial isolates. Specifically, we assess the performance of R10.4.1 flow cells in combination with the V14 version of the transposase-based (RBK) library preparation kit to provide rapid and accurate genomic epidemiological comparisons of bacterial species of clinical importance. We focused on retrospective collections of outbreak-associated *Corynebacterium diphtheriae* (CDIP) and vancomycin-resistant enterococci (VRE) and benchmarked expected performance parameters such as genome assembly quality, genotyping [multi-locus sequence typing (MLST) and core-genome multi-locus sequence typing (cgMLST)], SNP profiling and antimicrobial resistance and virulence prediction, against WGS data obtained routinely by Illumina MiSeq sequencing. Complete concordance with Illumina results was observed for MLST in both species and for cgMLST in CDIP, across all ONT kits and software evaluated. For VRE, however, cgMLST results varied with strain identity, library preparation kit and analysis parameters, likely due to software challenges to correctly call methylated bases. Yet, the use of the latest basecalling models combined with a PCR-based library preparation kit (RPB) reliably reproduced Illumina cgMLST results across all tested VRE strains. By testing two hybrid strategies combining PCR-free and PCR-based library preparation approaches, we also showed that combining PCR-free and PCR-based methods may yield a promising strategy, achieving both high accuracy and assembly completeness. Genomic-based antimicrobial resistance (AMR) prediction was consistent across sequencing methods, and we further highlight advantages and limitations of the PCR-based, PCR-free and mixed assemblies, to inform on the genomic context of AMR genes. This study demonstrates that a Nanopore-only sequencing approach may offer improved accuracy and consistency for classical bacterial typing in outbreak investigations, paving the way to wider use in clinical microbiology laboratories.

**Impact Statement**

This study demonstrates that an Oxford Nanopore Technologies-only sequencing approach improves the accuracy and consistency of bacterial typing in outbreak investigations, even for difficult cases such as vancomycin-resistant enterococci. This advancement paves the way for wider adoption of this technology in clinical microbiology laboratories, leading to more rapid and effective responses to bacterial infections.

## Data Summary

**Fig. 1.**
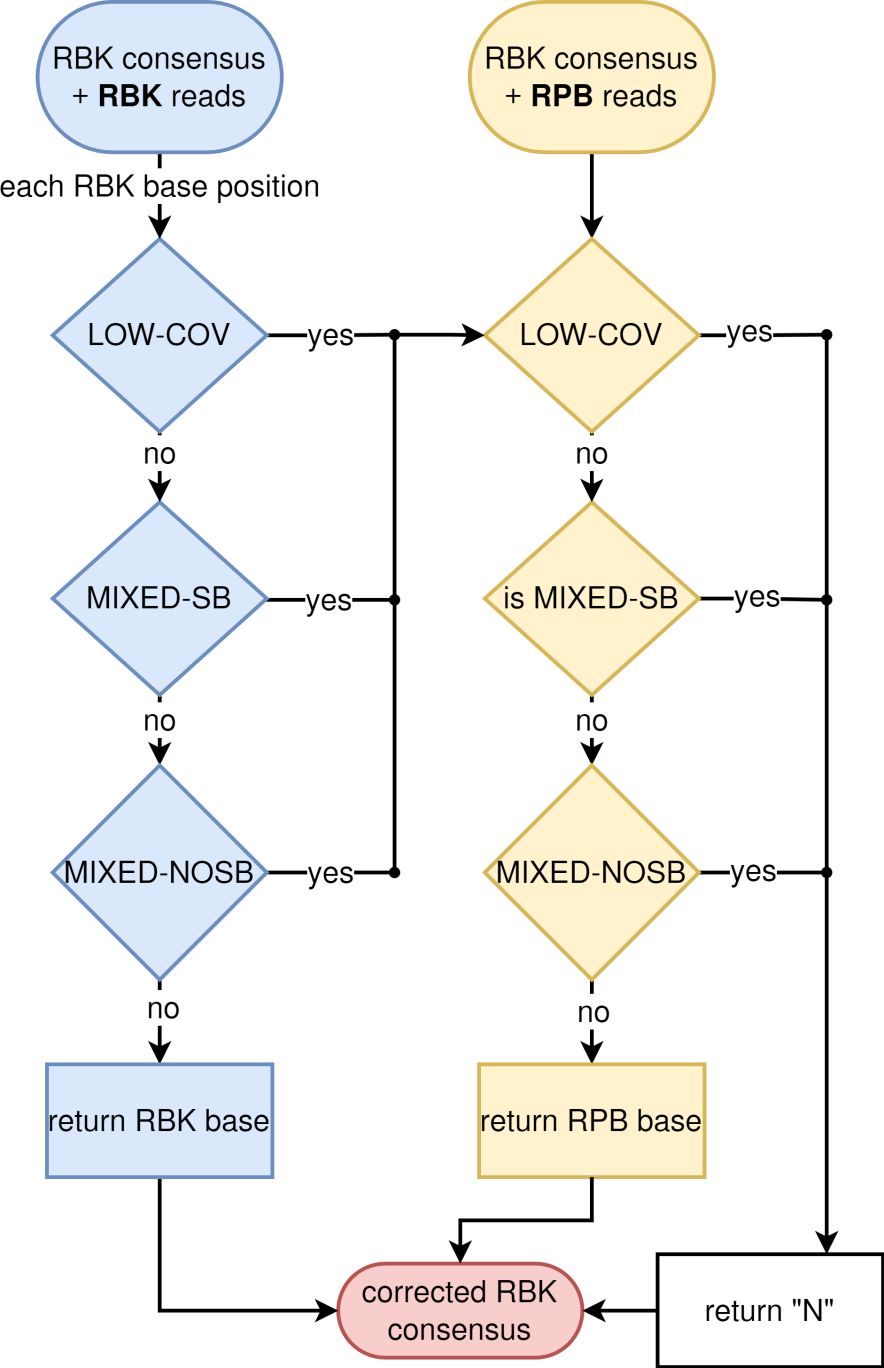
Correction algorithm of RBK-based assembly using RPB reads. Abbreviations: LOW-COV, coverage below the threshold of 20X; MIXED-SB, mixed base composition where the majority base constitutes more than 67% of all bases on one strand and less than 33% on the other strand; MIXED-NOSB, mixed bases that do not fulfil the criteria for strand bias mentioned above.

Illumina MiSeq and Oxford Nanopore Technologies (ONT) sequencing data for the Illumina, SUPD43, SUPDP43 and SUPD50 treatments are available under BioProject accessions PRJNA889706 and PRJNA1230056. NCBI SRA accession numbers for the individual libraries are listed in Table S2. ONT datasets generated using earlier basecaller versions are available upon request. The bioinformatic correction script (Fig. 1) is available at https://github.com/RametteLab/NanoporeHybridKP.

## Introduction

Whole-genome sequencing (WGS) has become the gold standard for bacterial strain typing, revolutionizing our ability to elucidate infection-epidemiological connections due to its high discriminatory power [1]. Consequently, WGS is increasingly employed in clinical settings to enhance outbreak detection, implement epidemiological surveillance and improve infection control, leveraging its unparalleled resolution [2,5]. Common approaches for assessing genetic relatedness from WGS data include gene-by-gene comparisons, such as core-genome multi-locus sequence typing (cgMLST) or whole-genome multi-locus sequence typing and SNP analysis [6]. While SNP typing offers high resolution, it is computationally intensive and time-consuming [7,8]. In contrast, cgMLST-typing provides a standardized nomenclature with lower computational demands [6]. Target-free, k-mer-based methods, like split k-mer analysis, are emerging but are not yet standard for transmission analysis [7,9]. Despite the widespread use of these methods, a standardized approach for interpreting WGS data in epidemiological investigations remains undefined [7].

Healthcare facilities have traditionally relied on short-read Illumina technology for WGS and genomic pathogen surveillance [10,11]. However, long-read next-generation sequencing (NGS), particularly provided by Oxford Nanopore Technologies (ONT) [12], is gaining traction. ONT sequencing offers rapid turnaround times, real-time data, long-read capabilities and lower capital costs, potentially reducing outbreak investigation times or enabling genomic surveillance in resource-limited settings [13,14]. Studies have shown promising results when using ONT sequencing for bacterial sequence typing and epidemiology [15,16], including largely consistent findings with Illumina for vancomycin-resistant enterococci (VRE) [17], *Mycobacterium tuberculosis* [18], *Staphylococcus aureus* [19] and methicillin-resistant *S. aureus* [20]. Several studies also demonstrate successful Nanopore-based typing in *Salmonella* [21,22], with one addressing homopolymer error reduction and combined Illumina/Nanopore analysis [22]. Smaller studies also report consistent results between Illumina and Nanopore for highly pathogenic bacteria [23], *Escherichia coli* [10], VRE [24], improved resolution of hospital VRE isolates [25] and *Klebsiella pneumoniae* strain typing [26].

Despite significant improvements in ONT technology, including higher throughput and lower error rates, and increasing use for bacterial genome assembly [27,28], challenges remain. These include the sensitivity of established typing methods like multi-locus sequence typing (MLST) [29,30], cgMLST or cgSNP [11] to single base errors potentially introduced by ONT sequencing. Consequently, there is a need to compare data generated with ONT to the more accurate Illumina sequencing for outbreak detection. For clinical diagnostics, where accuracy is key for correct treatment and control measures, these errors can be critical. This re-evaluation is particularly important given the recent improvement in ONT sequencing accuracy due to the development of newer flow cells and basecalling algorithms, making it increasingly reliable for clinical applications [23,31]. For instance, the R10.4.1 flow cell version has improved modal sequencing accuracy of 99.6% at the read level, which may be crucial for high-resolution genotyping necessary in clinical diagnostics. In addition, ONT transposase-based library preparation kits (e.g. RBK) now offer very fast turnaround time from isolates to sequencing results. Further, the possibility to combine PCR and PCR-free library preparation has not been fully assessed in terms of turnaround time, cost and effects on the genomic sequencing accuracy.

To validate ONT V14 kits for genomic epidemiology in outbreak settings, we performed a retrospective, comparative study using clinical isolates of *Corynebacterium diphtheriae* (CDIP) and VRE, which we obtained from our routine diagnostic laboratory. Beyond their clinical relevance, these isolates were selected because they were managed through a fully integrated workflow spanning isolation, storage, DNA extraction, sequencing and bioinformatic analysis conducted by the same expert team. This approach ensures our evaluations closely replicate real-world clinical diagnostic settings and methodological consistency. Recognizing that Illumina short-read sequencing may represent the accuracy gold standard, but may also be limited in resolving genomic structures, our goal was to determine if ONT long reads provide actionable data for outbreak decision-making. We focused on evaluating ONT performance in key areas: typing (MLST and cgMLST), antimicrobial resistance (AMR) and plasmid analysis in these two bacterial outbreak examples.

## Methods

### Sample origin, cultivation and DNA extraction

All CDIP isolates were associated with local outbreaks in Switzerland from July to September 2022 and were described in a previous study [32]. The corresponding Illumina MiSeq data are available as BioProject PRJNA889706. VRE isolates, associated with local hospital outbreaks in a Swiss hospital, were collected between 2017 and 2021, with some of them belonging to ST796 [33]. They were isolated on CHROMagar VRE plates (CHROMagar, Paris, France) during routine analysis by the clinical microbiology laboratory of the Institute for Infectious Diseases (IFIK), University of Bern. Species identification was determined by MALDI-TOF (Bruker Daltonics, Bremen, Germany). Susceptibility testing for all isolates was performed during routine clinical processing at IFIK. All isolates were stored at −80°C and re-grown on CSBA plates before performing genomic extraction, thus minimizing the risk of *in vitro* evolution. Genomic DNA was extracted from agarose plates using PureLink Genomic DNA kit (Thermo Fisher, Switzerland), or with Maxwell RSC Cultured Cells DNA Kit (Promega, Switzerland).

### Whole-genome sequencing

NGS libraries for Illumina sequencing were produced with the Nextera DNA Flex Library Prep Kit (Illumina, Switzerland), according to the manufacturer’s recommendations, and sequenced on an Illumina MiSeq sequencer with v2 reagents in 2×150 paired-end mode, at the Next Generation Sequencing Platform of the Institute for Infectious Diseases, Bern. Nanopore sequencing libraries were produced with the rapid barcoding chemistry SQK-RBK114.96 (RBK) and Rapid PCR barcoding chemistry SQK-RPB114.96 (RPB) according to the manufacturer’s recommendations. The libraries were loaded onto standard GridION flowcells (FLO-MIN114) and sequenced in batches of 21 samples for 12–72 h on GridION X5 sequencers with real-time basecalling and under high-accuracy mode (HAC). The raw signal files (fast5_pass/pod5_pass directories) were subsequently re-basecalled either on the GridION or with standalone basecallers on a separate Linux workstation. All software versions and basecaller models are provided in Table S1 (available in the online Supplementary Material).

### Ethics statement

All CDIP and VRE isolates in the present study have been anonymized, and no patient information is used in the interpretation of the bacterial genomic data. Publication of this analysis does not harm or influence either patients or institutions. Ethical committee approval was, therefore, not requested.

### Bioinformatic analyses

**Fig. 2.**
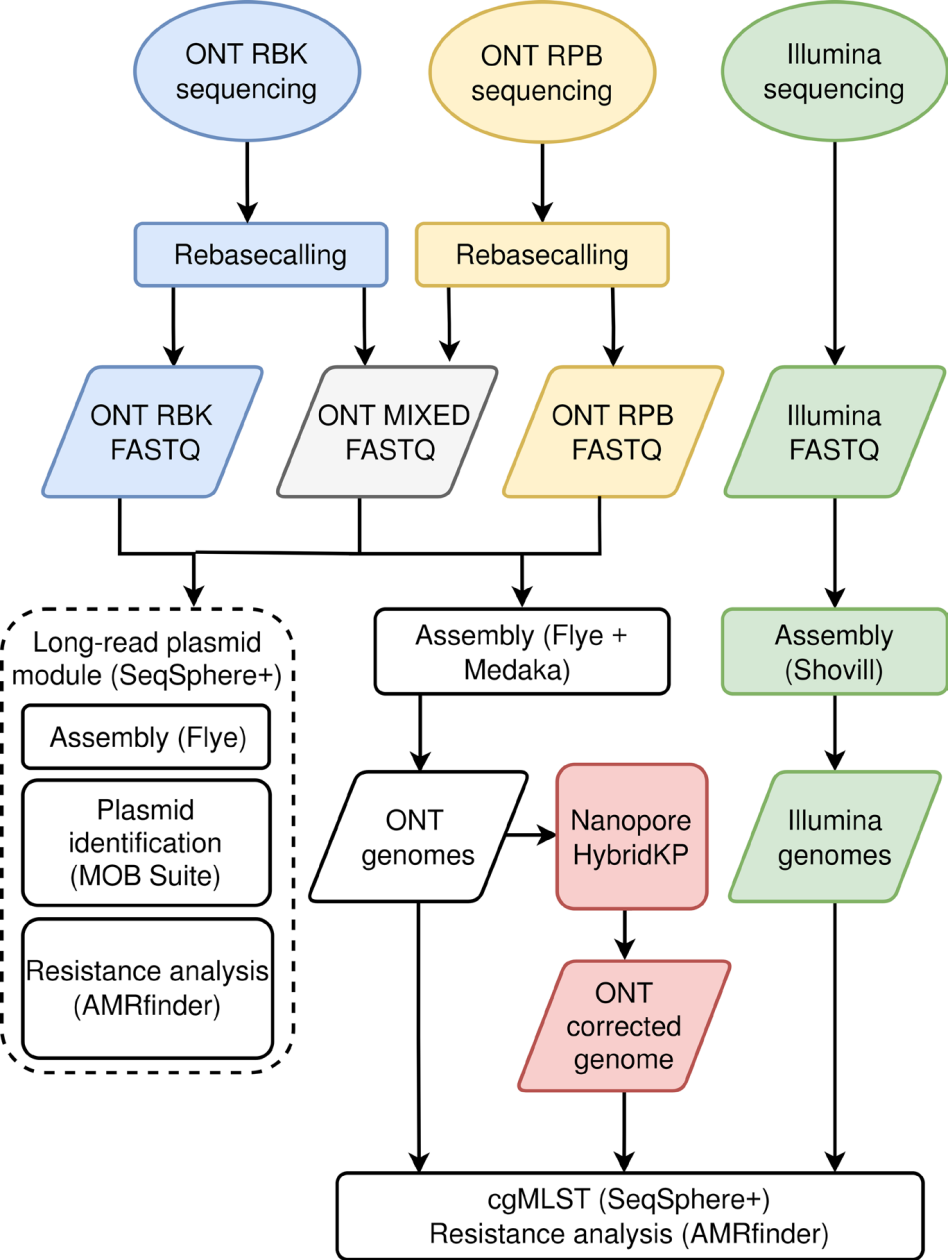
Overview of the bioinformatic analyses performed in the study.

*Genome assembly and polishing*. All genomes were reconstructed with the same software versions and parameters, except for the assembly parameters and for the models used for genome polishing, which were adjusted to the basecalling mode (SUP or HAC), and the basecalling model, respectively (Fig. 2). All reads were filtered to remove sequences shorter than 500 bases and those with an average quality score below 10. In addition, 30 bases were trimmed from both the 5′ and 3′ ends of each read to remove lower quality bases (*NanoFilt*, version 2.8.0, parameters: -q 10 -l 500 --headcrop 30 --tailcrop 30) [34]. The remaining reads were assembled with *Flye* (version 2.9.2) [35], and polished with *Medaka* (version 1.11.3) [36]. Detailed information, including the sequencing and basecalling software, basecalling models, assembly parameters and Medaka models, can be found in the supplementary material (Table S1). To mask bases with ambiguous base compositions in the corresponding reads, selected assemblies were processed with the software MPOA [37].

*Combining PCR-based and PCR-free Nanopore reads*. We tested two different approaches to combine RBK and RPB data: (a) Read mixtures. Random subsets of the reads produced with RBK and RPB were combined at theoretical coverage depths of 70× and 30×, respectively, to obtain mixed datasets with total coverage depths of 100×. These read sets were processed as described above for the non-mixed datasets. (b) Assembly correction. Reads sets produced with RBK and RPB were mapped separately against an assembly produced from the RBK reads with the software *minimap2* (version 2.24) [38]. The resulting SAM files were sorted (*samtools*, version 1.15.1) [39] and further analysed with the software *Pysamstats* (version 1.1.2) [40] to obtain per-strand base compositions for each assembly position. Base composition statistics were then processed using a custom Python script as follows (Fig. 1): (1) We assigned a confidence level (high or low) to each base of the original RBK assembly, based on the underlying base compositions of the mapped reads. Assembly positions were considered as ‘low confidence’ if one or more of the following criteria applied: Low read coverage (<20× coverage depth), mixed base composition (<80% of the reads matching the most frequent base) or strand bias (different dominant bases between the forward and reverse strand). Otherwise, the positions were considered as ‘high confidence’. (2a) The RPB majority consensus base was obtained for each base position of the original assembly. (2b) We assigned a confidence level (high or low) to the RPB majority consensus bases, using the same criteria described in step one above. (3) The assembly was corrected for low confidence positions in the original assembly, which were replaced by the majority consensus of the RPB reads if the confidence was high for the latter and, by N, if the confidence in the RPB read set was low too. Each ‘high confidence position’ of the original assembly was kept.

*Typing and comparison of the different treatments*. All assemblies were imported into *SeqSphere*+ (v.8.4.0; Ridom GmbH, Germany) and processed with the cgMLST schemes *Enterococcus faecium* cgMLST v1.1 [41] and a custom task template encompassing 1,319 core genes for CDIP based on the Institute Pasteur scheme, as described in [32]. Comparison tables (containing allele IDs for each target gene and assembly) were created for the Nanopore and Illumina assemblies of each analysed species and exported for further processing with custom Python and R scripts.

*AMR and plasmid analyses*. Protein sequences were predicted with the software *Prokka* (version 1.14.6) [42] and used as input for *AMRFinderPlus* (version 3.11.2, database: 2023-08-08.2, parameters -p -g -n --annotation_format prokka --organism) [43]. For plasmid analysis, WGS reads from treatments ‘SUPD43’, ‘SUPDP43’ and ‘SUPD and P43’ were reassembled and analysed with the ‘long read’ module with Flye as assembler and default parameters (*SeqSphere*+, version 10.0.0). Comparison tables including the task template fields ‘Chromosome and plasmid Overview’, as well as a local plasmid database (created via ‘Create Task Template for Plasmid Mash Database’), were generated to facilitate data extraction from SeqSphere+. Assembly statistics (number of replicons, circularity) and plasmid-borne resistance were extracted from the resulting output tables and further analysed using custom Python scripts.

## Results

### VRE genomic epidemiology using Nanopore vs. Illumina WGS

**Fig. 3.**
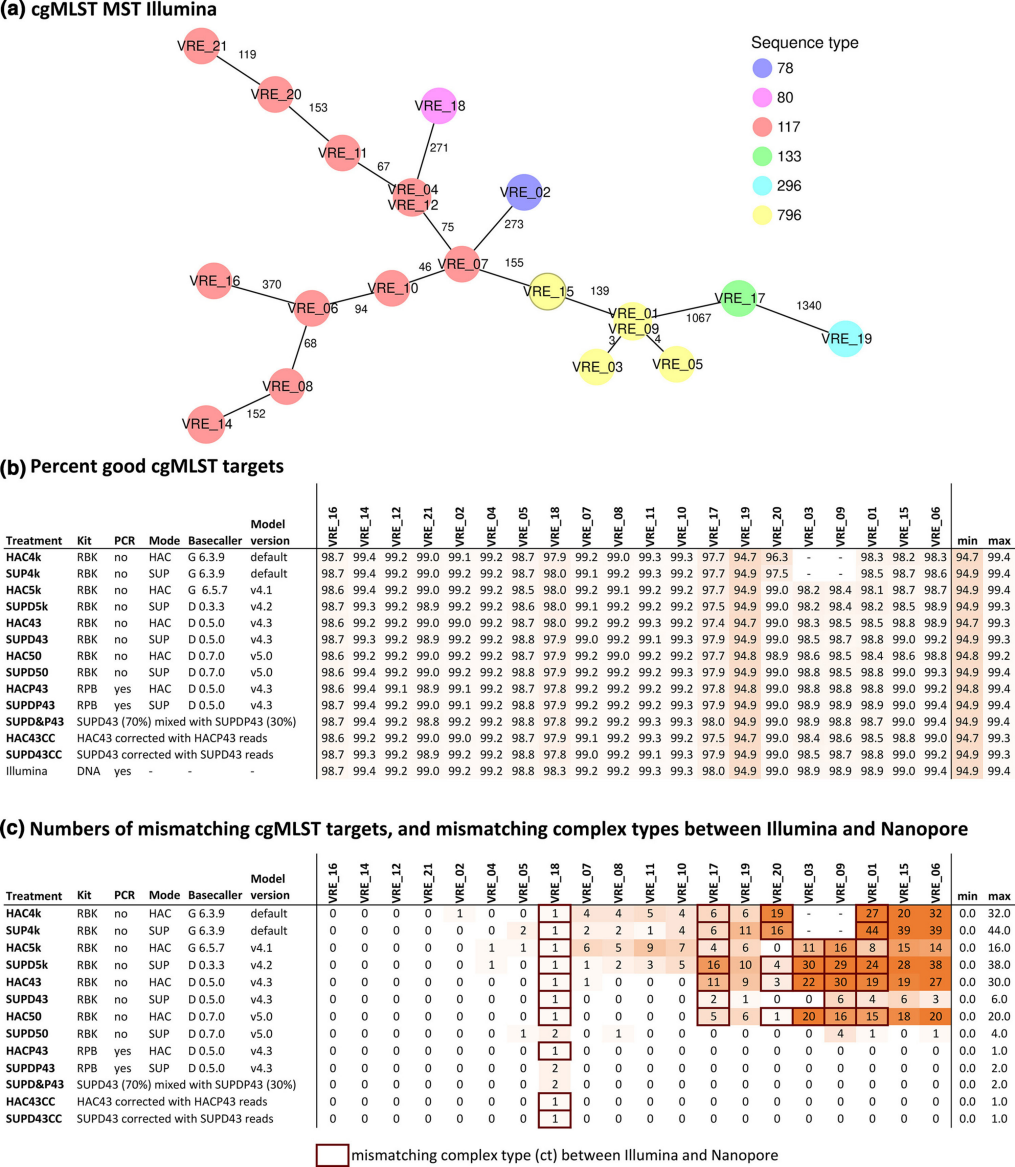
Comparison of cgMLST results for clinical VRE isolates sequenced with Illumina or Nanopore. The cgMLST scheme contained 1,423 target genes. (**a**) Minimum spanning tree of the 20 Illumina assemblies. (**b**) Percentage of the 1,423 cgMLST target genes that were detected (and passed the QC) in the different assemblies. (**c**) Number of mismatching alleles between the ONT and corresponding Illumina assemblies, and mismatching complex types between the two technologies (indicated by red boxes). Letters G and D in the column Basecaller stand for Guppy and Dorado, respectively. A detailed explanation about treatments is provided in Table S1.

We choose a representative and diverse collection of clinical VRE isolates, consisting of 6 different MLST sequence types, 3 clonal clusters and 12 different cgMLST complex types based on Illumina assemblies (Fig. 3a). In terms of target gene recovery, we observed near-identical performance between Illumina and the top-performing Nanopore WGS methods: Percentages of successfully detected cgMLST target genes in the assemblies were highest for Illumina (98.80%), closely followed by the PCR-based Nanopore approach SUPDP43 (98.77%) and the most recent PCR-free approach SUPD50 (98.71%). The lowest percentage was found for the HAC43.masked (97.46%), which was still well above the quality threshold of 90% (Fig. 3b).

A near-perfect agreement was found between Illumina and the top-performing Nanopore methods for the individual cgMLST allele assignments. The numbers of mismatching alleles between the Illumina and Nanopore assemblies varied strongly between isolates, Nanopore software versions, basecalling models and the library preparation kits: For VRE21, for example, no mismatching alleles were detected, irrespective of the approach used to create the Nanopore assemblies, whereas 0 to 44 mismatching alleles were detected in the assemblies produced from isolate VRE01 (Fig. 3c). Noticeably, PCR-based library preparation outperformed PCR-free alternatives irrespective of the basecalling models used, allowing for a perfect replication of the allelic profiles obtained with Illumina in all but one case, with a maximum number of two mismatches (VRE18). Mixtures of PCR-based and PCR-free reads, as well as PCR-free assemblies that were corrected with PCR-based reads, performed on par with PCR-free reads in these aspects.

The PCR-free approach was also more sensitive to the analysis parameters: SUP models clearly outperformed HAC models, and the most recent iterations of the software (and models) outperformed previous versions. The best combination of parameters resulted in perfectly matching allele assignments in 14 out of 20 cases with a maximum of four mismatches (Fig. 3c). Subsequent masking with the software *MPOA* resulted in the exclusion of all but one of the previously mentioned mismatching alleles (*SeqSphere*+ excludes genes with N’s from the analysis), while the percentage of successfully detected cgMLST targets dropped by 0.8%, remaining well above the threshold of 90% (SUPD34, 98.68%; SUP43.masked, 97.90%, Fig. S1A, B).

MLST classification into sequence type (ST) was successful across all tested combinations that did not involve masking with MPOA (Fig. S1D). The latter resulted in two unassigned STs in the treatment HAC43.masked (Fig. S1D), whereas treatment SUP43.masked did not result in a missing ST assignment. cgMLST classification into complex type (CT) was more sensitive to the variability introduced by the different treatments: The PCR-based treatments SUPDP43 and HACP43 matched the Illumina CT in all cases and in 19 out of 20 cases, respectively. The single CT 1552 (instead of CT 2887) that differed between the PCR-based treatments was a result of one target gene (EFAU004_01770, hypothetical protein), which was exclusively found in SUPDP43 in otherwise identical allelic profiles (Fig. S1D). The mixtures of PCR-based and PCR-free reads matched the Illumina CT in all cases and the PCR-free assemblies that were corrected with PCR-based reads in 19 out of 20 cases, respectively. The single mismatch was caused by the absence of the same target gene, as described above for the PCR-based approaches. Among the PCR-free treatments, SUPD50 performed the best with all 20 correct CTs assigned, followed by SUPD43 with 17/20 correct assignments (Fig. 3c). Subsequent MPOA masking (SUP43.masked) corrected two of the remaining mis-assigned CTs, but another CT was assigned for VRE10, while no CT was assigned for it based on the Illumina assembly (Fig. S1).

**Fig. 4.**
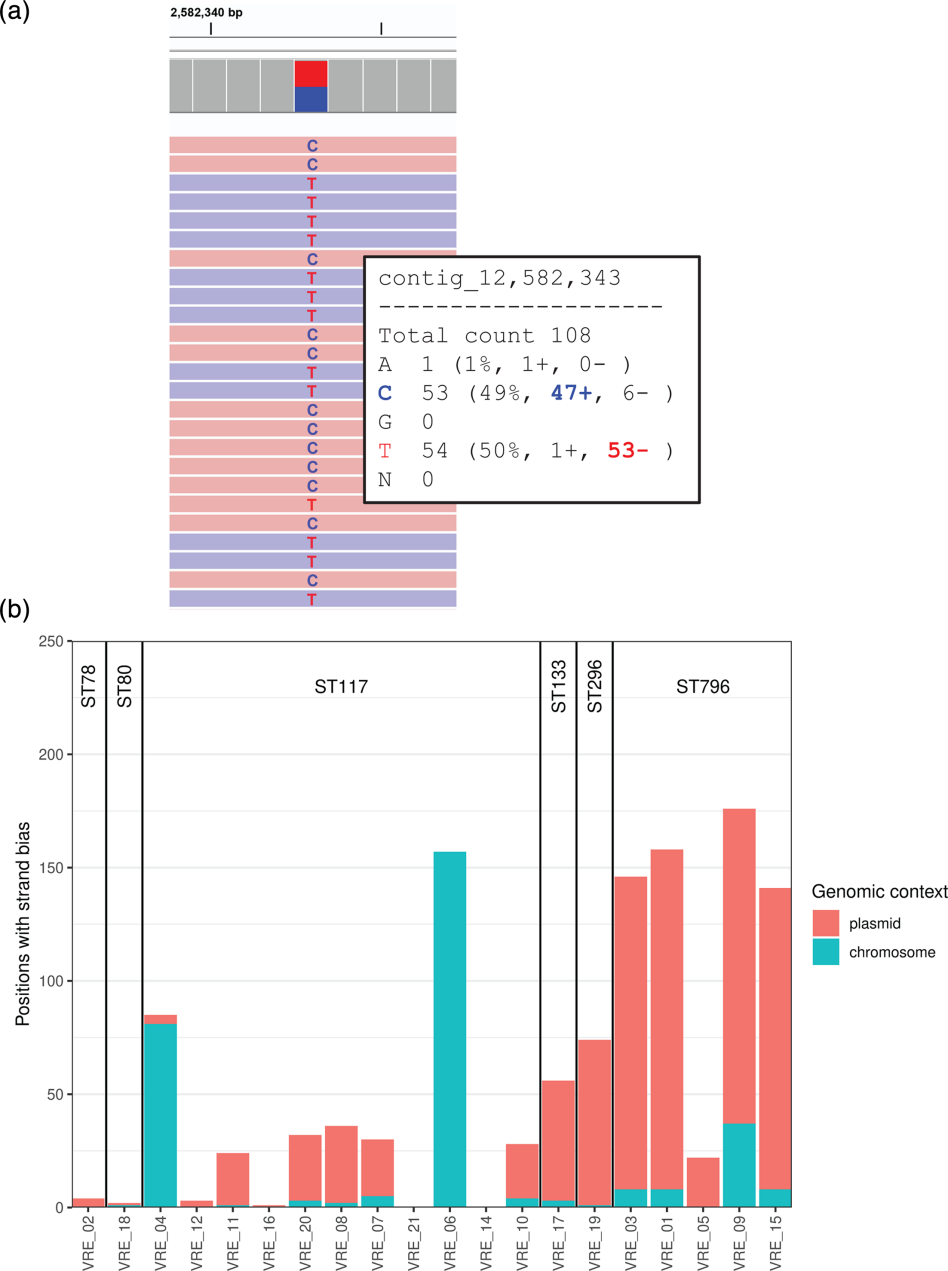
**(a**) Example of an ambiguous base position: The majority of forward and reverse reads differ at the called base. Strand direction is indicated by read color. (**b**) Numbers of ambiguous base positions detected by genomic locations and by MLST ST.

Noticeably, a large proportion of the mismatching bases between Illumina and Nanopore assemblies were found in assembly positions with conflicting information between the forward and reverse reads (Fig. 4a). This phenomenon is referred to as strand bias (SB) in subsequent sections. We analysed the frequency of SB as a function of its genomic context and MLST types (Fig. 4b). In our dataset, SB was more often observed on plasmids than on chromosomes. No distinct pattern related to ST was identified; instead, SB levels appeared to be strain-specific rather than ST-specific (Fig. 4b).

### VRE-predicted AMR and plasmids

**Fig. 5.**
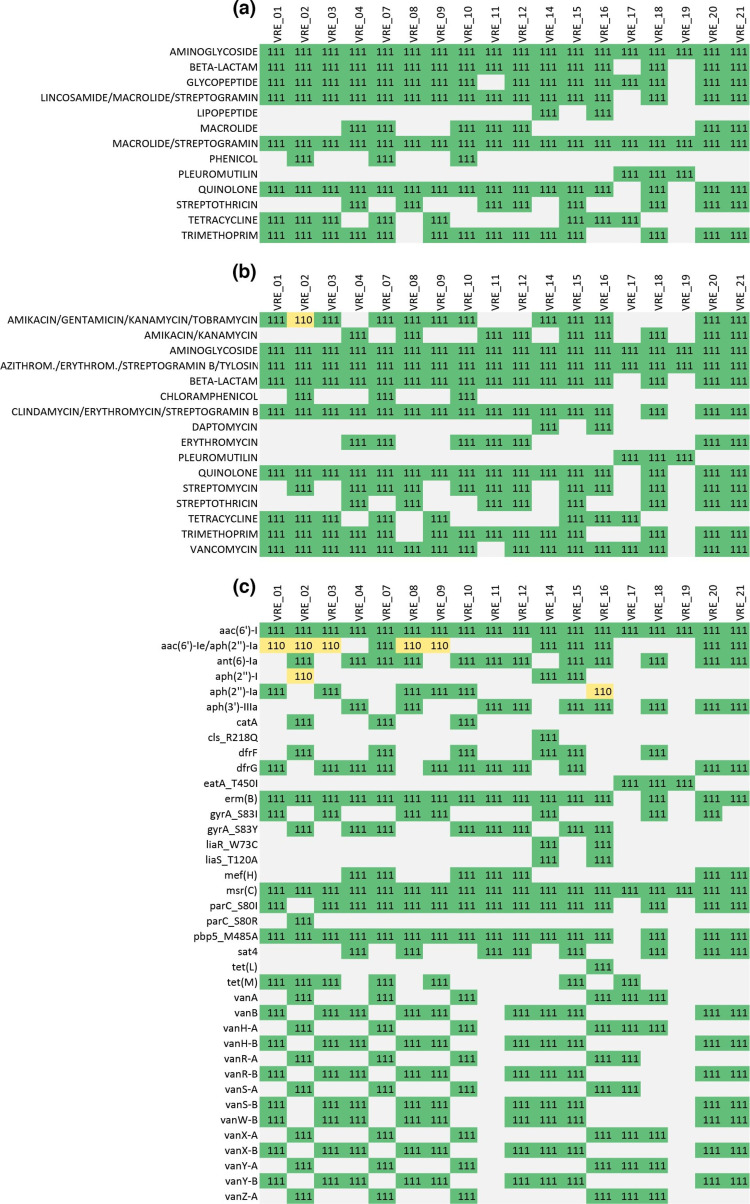
Comparison of the predicted resistance genes based on ONT and Illumina assemblies. Tuples indicate presence (1) or absence (0) in the treatments SUBDP_43 (RPB), SUPD_43 (RBK) and ILLUM (Illumina) with (a) antibiotic classes, (**b**) antibiotic subclasses, (**c**) resistance genes.

**Fig. 6.**
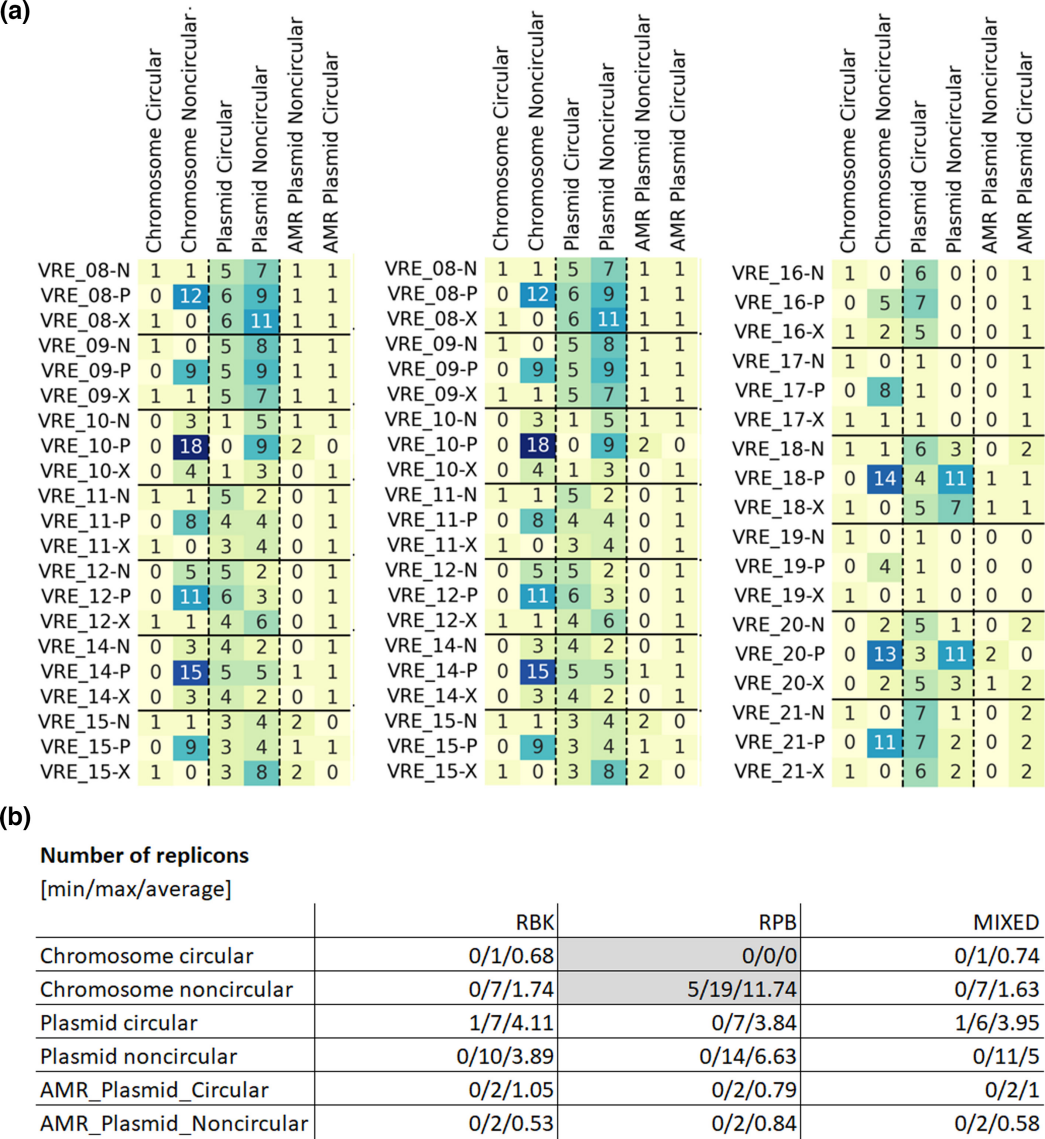
Assembly performance of PCR-based, PCR-free and mixed Nanopore reads. (**a**) Numbers of contigs by categories based on replicon type, circularity and presence of AMR genes. (**b**) Summary of the data provided in (a). Grey shadings indicate significant differences to the other treatments within one category (Mann-Whitney *U* test; corrected *P*-value for multiple testing, *P*<0.05).

To evaluate whether ONT assemblies are suitable for AMR analysis, we applied AMRFinderPlus to the Illumina and ONT assemblies: On the class level, predictions for the Illumina and ONT-based assemblies were matched in all cases (Fig. 5a). On the antibiotic subclass level, discrepancies were found for one isolate (VRE_02), for which gentamicin and tobramycin resistances were exclusively predicted for the ONT assemblies (Fig. 5b). On the gene level, the bifunctional aminoglycoside-modifying enzyme *AAC(6′)-Ie–APH(2′′)-Ia*, which confers resistance to all commercially available aminoglycosides except streptomycin [44], was exclusively detected in the ONT assemblies in five cases (Fig. 5c). However, in all but one isolate (VRE_02), the C-terminal domain of *AAC(6′)-Ie–APH(2′′)-Ia* was detected in the Illumina and ONT assemblies, explaining the matching resistance predictions (Fig. 6a, b).

Different Nanopore approaches were compared with respect to their performance for plasmid analysis (PCR-free, PCR-based and mixtures of both). PCR-free and mixed Nanopore methods showed superior chromosome assembly performance than PCR-based Nanopore assemblies, as PCR-based Nanopore assemblies showed a higher degree of chromosome fragmentation than PCR-free or mixed assemblies. Plasmid assembly performance did not differ significantly between the tested methods (Fig. 6).

**Fig. 7.**
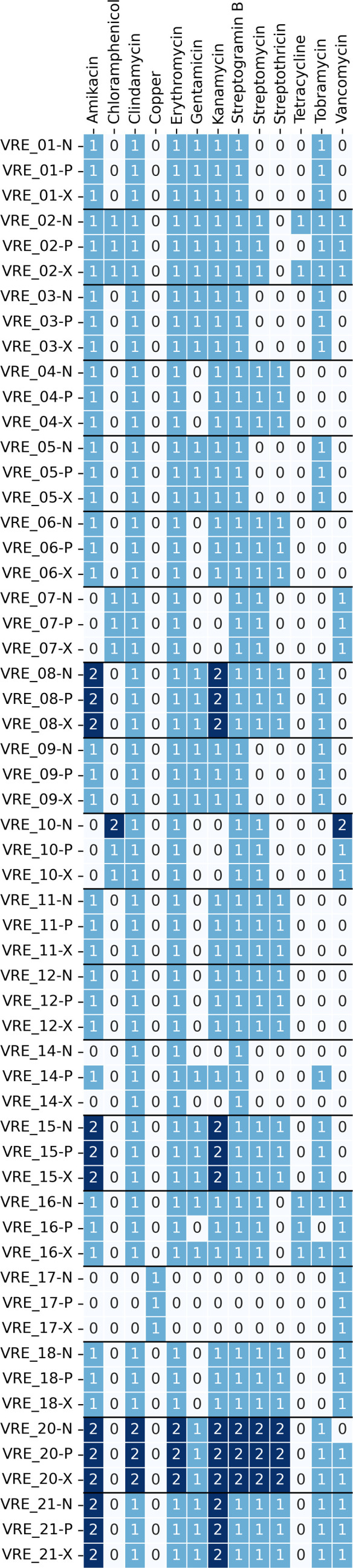
Plasmid-borne resistance. Numbers indicate the number of resistance genes detected for each specific antibiotic subclass.

Discrepancies in the predicted genomic context of resistance genes were identified in 4 out of the 19 isolates when comparing the tested Nanopore approaches: Tetracycline resistance in VRE02, along with gentamicin and tobramycin resistance in VRE16, was determined to be plasmid-mediated in PCR-free and mixed assemblies, but not in PCR-based assemblies. Conversely, amikacin, gentamicin, kanamycin and tobramycin resistance in VRE14 were identified as plasmid-mediated only in the PCR-based assemblies. Vancomycin resistance was predicted to be plasmid-mediated in PCR-based and mixed assemblies of VRE20, but not in the PCR-free variant (Fig. 7).

### CDIP genomic epidemiology using ONT vs. Illumina WGS

**Fig. 8.**
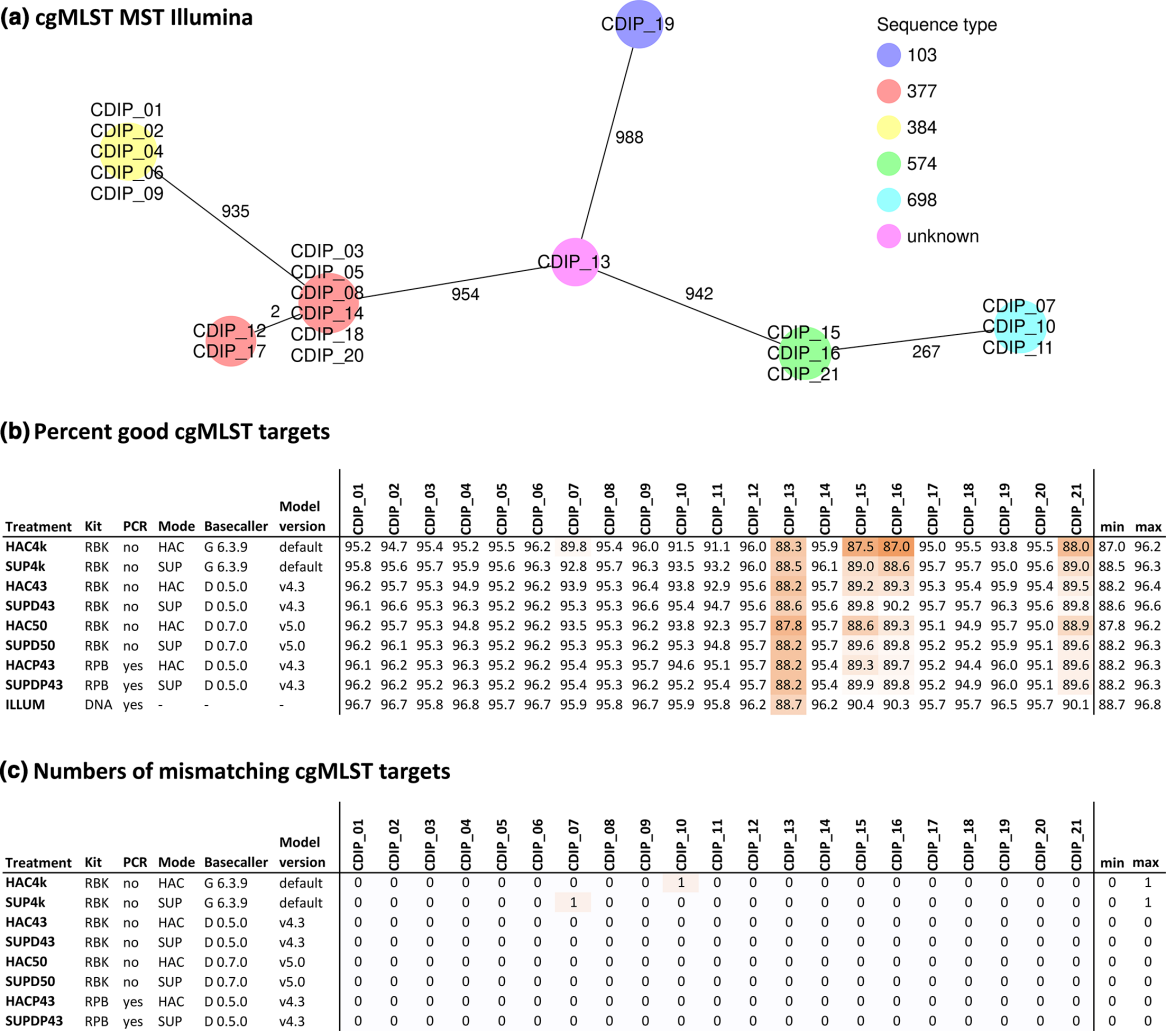
Comparison of cgMLST results from 21 clinical *C. diphtheriae* isolates sequenced in parallel with Illumina and ONT. The cgMLST scheme contained 1,312 target genes. Treatment: Sequencing and data processing protocol used (Table S1). (**a**) Minimum spanning tree of the 21 Illumina assemblies. (**b**) Percentage of the 1,312 cgMLST target genes that were detected (and passed the QC) in the different assemblies. (**c**) Numbers of mismatching alleles between the ONT and the corresponding Illumina assemblies.

Similarly, a high level of diversity was expected in the chosen collection of clinical CDIP isolates with six different MLST sequence types and five clonal clusters based on Illumina assemblies (Fig. 8a). Percentages of successfully detected cgMLST target genes in the assemblies were highest for Illumina (94.96% on average), but on par with the Nanopore approach SUPD50 (94.38% on average) (Fig. 8b). Masking of ambiguous base positions reduced the number of successfully detected cgMLST target genes by 1.1 and 1.5% in the treatments SUPD43.masked and HAC43.masked as compared to their unmasked counterparts SUPD43 and HAC43, respectively (Fig. S1). Between Illumina and all tested Nanopore approaches, individual alleles were assigned in perfect agreement for MLST (Fig. S2C), and with very high agreement for cgMLST, with a maximum of one differing allele per Nanopore approach (Fig. 8c). The AMR predictions for CDIP showed complete concordance between Illumina and ONT assemblies at the class level (Fig. S3A). However, a single discrepancy was observed at the sub-class and gene levels for two isolates (CDIP_05 and CDIP_18). Specifically, the aminoglycoside phosphotransferase gene *aph(3′)-Ia*, conferring kanamycin resistance, was absent in the PCR-based ONT assemblies of both isolates (Fig. S3B, C).

### Time and cost estimation

The ONT approaches used in this study are competitive with Illumina both in terms of hands-on time, overall turnaround time and costs per sample at the tested throughput levels (Table 1). For example, preparing 20 samples takes only 0.7 h (RBK) or 1 h (RPB) hands-on time, significantly less than the 1.8 h required for Illumina MiSeq. The overall turnaround time is also notably faster for smaller batches: For four samples, ONT (RBK or RPB) finishes sequencing in 5 h, while Illumina requires 24 h. There is a slight cost increase when combining ONT RBK with RPB workflows, as the latter involves the use of long-range PCR in addition to the transposase library preparation as in the RBK protocol. Most critically, the cost reduction is substantial: For batches of 20 samples, the cost is estimated at $310 (RBK) or $400 (RPB), compared to $2,340 for the MiSeq run, leading to a cost difference per sample of $117 for MiSeq vs. $36 for the combination of RBK and RPB on the same sample, so a 3.25-fold cost reduction. This significant cost reduction can be obtained given the possibility to wash ONT flow cells with a DNase treatment and to stop the sequencing run earlier when enough reads have been obtained, as compared to the Illumina-based approach, which entails fixed costs and run durations (Table 1).

**Table 1.** Turnaround time and cost estimates

	Illumina MiSeq	ONT RBK*	ONT RPB†
Library preparation (hands-on/total)			
*n*=4 samples	1.5/2.7 h	0.5/1.0 h	0.7/3.0 h
*n*=20 samples	1.8/2.7 h	0.7/1.2 h	1.0/3.3 h
Sequencing time			
*n*=4 samples	24 h	5 h	5 h
*n*=20 samples	24 h	24 h	24 h
Cost estimates per batch (per sample)‡			
*n*=4 samples	$468 ($117)	$200 ($50)	$300 ($75)
*n*=20 samples	$2,340 ($117)	$310 ($16)	$400 ($20)

*ONT transposase-based library preparation (RBK).

†ONT transposase-based followed by PCR-based library preparation (RPB).

‡Costs are estimated at $117 per sample for MiSeq based on 20 isolates per run, excluding personnel costs. For ONT runs, we hypothesized conservatively that 24 h and 5 h runs correspond to half and a fourth of the maximal lifespan of a flow cell, whose total cost is estimated at $500. The costs are indicative and may vary depending on the country and specific contract with the involved companies.

## Discussion

We assessed the recovery of cgMLST target genes, allele assignments, MLST and cgMLST classification, prediction of AMR genes and plasmid content using various Nanopore library preparation methods (PCR-based, PCR-free and mixtures) and analysis pipelines. We evaluated a diverse, clinically relevant collection of outbreak isolates, originating from local outbreaks, which were classified as different MLST sequence types and clonal clusters based on Illumina WGS assemblies. Our main findings indicate that MLST classification was generally successful across Nanopore approaches, while cgMLST CT classification showed more sensitivity to the different Nanopore approaches. We also explored the suitability of Nanopore assemblies for AMR analysis, revealing generally concordant results with Illumina at the class level for AMR but some discrepancies at the subclass and gene levels. The nanopore workflows that performed best in cgMLST were further compared with respect to their ability to predict the genomic context of resistance genes using the SeqSphere+ Long read Data Plasmid Transmission Analysis Module. This analysis yielded largely consistent results, with only a few discrepancies. We selected this approach because it provides a straightforward framework for plasmid characterization and demonstrated higher consistency than a plasmid analysis in SeqSphere+ based on the preassembled contigs (Figs S4 and S5). Finally, the study provided turnaround time and cost estimation, suggesting that the tested Nanopore approaches are competitive with those offered by short-read NGS approaches. With more than 550 resulting assemblies analysed with MLST and cgMLST to determine clonal complexes and accuracy of the epidemiological conclusions, we highlighted how type of WGS data produced combined with the software running on Nanopore sequencers, as well as parameters selected by the operator, can have a major influence on the quality of the resulting sequencing reads and the epidemiological conclusions derived from the WGS data. Our results have practical consequences for genomic studies that aggregate sequencing data originating from different laboratories using different technologies or software versions.

While Nanopore demonstrated near-identical performance to Illumina for cgMLST target gene recovery and MLST typing, some discrepancies were observed in cgMLST allele assignments and AMR gene detection depending on the specific Nanopore approach used. Notably, PCR-based Nanopore library preparation generally outperformed PCR-free methods for allelic profiling in VRE. We concluded that optimized Nanopore sequencing and analysis pipelines offer a competitive alternative to Illumina for genomic surveillance of these pathogens in terms of accuracy, time and cost. Complete concordance with Illumina results was observed for MLST in both tested species and for cgMLST in CDIP across all ONT kits and software evaluated. In contrast, the accuracy of cgMLST results for VRE varied based on the strain, library preparation kit and analysis parameters, likely due to challenges in resolving base methylations. The latest software (≥Dorado 0.5.0) and basecalling model versions (≥v4.3), combined with the PCR-based library preparation kit (RPB), reliably reproduced Illumina cgMLST results across all tested VRE strains.

Previous studies assessing ONT performance for accurate genome reconstruction often did not use the latest generation of flow cells, sequencing kits and software. For instance, Foster-Nyarko *et al.* [45] found R9.4.1/V10 chemistry reliable for MLST and AMR in *K. pneumoniae*, but cgMLST/SNP cluster detection remained challenging. Greig *et al*. [10] noted 95% of nt discrepancies between Illumina and ONT data for two isolates of Shiga toxin–producing *E. coli* (STEC) O157:H7, which could be resolved by masking methylated and prophage regions in ONT data. More recent R10.4/V12 studies showed mixed results: [13] found V12 chemistry accuracy promising but throughput insufficient for large-scale studies. [23] found high-resolution cgMLST/cgSNP genotyping feasible for *F. tularensis* and *B. anthracis*, but not *B. suis.* [28] concluded R10.4 cells yield ‘near-finished’ bacterial genomes without short-read polishing, though they did not test high-resolution typing. Conversly in [14], V12 chemistry accuracy was found to be sufficient for *Bordetella pertussis* clinical epidemiology via cgMLST. Despite V12’s accuracy improvements, especially for homopolymer errors, previous generations were limited by reduced data throughput. The latest R10.4.1/V14 chemistry was evaluated by [37] in a *K. pneumoniae* outbreak using cgMLST, concluding that ONT sequencing caused considerable base errors leading to incorrect exclusions in outbreak tracing. They suggested mitigating these methylation-related errors through PCR-based library prep or masking ambiguous base positions.

Our study thus expands the knowledge on ONT performance for WGS-based bacterial typing by applying a similar comparative framework to two sets of well-characterized outbreak strains from the highly relevant species CDIP and VRE, both with native and PCR-based library preparation kits. In contrast to previous studies, we put a strong focus on the influence of different sequencing and basecalling software on the accuracy of downstream analyses such as cgMLST and AMR gene contextual analyses. Thus, we tested multiple software versions released in 2023 and 2024, in combination with different parameters such as different versions of the GridION software, varying sampling rate of the signal acquisition (4 khz and 5 khz), basecaller software (Guppy and Dorado), basecaller versions and basecalling models. All sequencing runs were conducted with real-time basecalling in HAC. The acquired signals were later re-basecalled in HAC and SUP mode, with standalone versions of the basecallers Guppy and Dorado, and different basecalling models. Our study thus reflects and integrates in its evaluation the rapid evolution of the technology over the past 2 years, including both wet laboratory and software changes that initially reduced the accuracy of the tested applications. We show the significant influence such an update may have on ONT datasets when processing native DNA libraries. Further strengths of our study are that we also provided estimates of both turnaround time and associated costs to offer a comprehensive perspective on implementation considerations.

Our study also identified several weaknesses and areas where ONT sequencing showed variability or discrepancies compared to Illumina. The number of mismatching alleles between Illumina and Nanopore assemblies varied strongly depending on the isolate, ONT software versions, basecalling models and library preparation kits. PCR-free library preparation was found to be more sensitive to analysis parameters than PCR-based methods. Masking of ambiguous base positions with MPOA could lead to unassigned STs and a reduction in successfully detected cgMLST target genes. Discrepancies were observed in AMR predictions at the antibiotic subclass and gene levels for certain isolates. Furthermore, the predicted genomic context of resistance genes (chromosomal vs. plasmid-mediated) showed discrepancies between different Nanopore approaches in some cases. However, we cannot exclude that optimizations in the plasmid reconstruction procedure could improve the consistency between different nanopore approaches [46], but this was outside the scope of the study.

Despite its strengths, the PCR-based ONT kit exhibited certain drawbacks, such as a smaller read length, ultimately leading to more fragmented chromosome assemblies. To mitigate this, we tested two hybrid strategies combining PCR-free and PCR-based Nanopore approaches and demonstrated promising results. These combined methods indeed retained the accuracy of the PCR-based approach while achieving assembly performance comparable to PCR-free methods. AMR prediction at the genome level was consistent across all tested sequencing methods; however, resolving the genomic context of AMR genes remains a complex task [47]. Although ONT long-read sequencing may offer significant improvements in determining genomic context compared to short-read technologies, we did observe inconsistencies in the predicted genomic context of selected AMR genes when comparing datasets produced from PCR-based, PCR-free and mixed Nanopore reads. As we chose a retrospective collection of outbreak-related isolates for in-depth comparative analyses, future work would need to provide a prospective evaluation to assess the performance of the new technology in actual clinical or epidemiological settings, thus offering a more accurate reflection of real-world applicability. Also, we limited ourselves to local outbreaks, for which the source of material and reference short-read data were controlled. We may also ask how international collection and inter-laboratory comparison would perform in such a comparative framework.

The results of this study suggest that Nanopore WGS is a promising technology for genomic epidemiology of outbreak bacterial species, such as VRE and CDIP, demonstrating comparable performance to Illumina in key areas such as cgMLST target gene recovery and allele assignment. The strengths in chromosome assembly using PCR-free methods and the competitive time and cost further support this potential. The observed variability related to software and parameter specification and the discrepancies in AMR and plasmid analysis highlight the need for continued optimization of Nanopore sequencing protocols, basecalling algorithms and analysis pipelines to improve accuracy and reliability in genomic epidemiological applications. Future research could focus on refining PCR-free methods to reduce their sensitivity to analysis parameters, developing more robust methods for resolving ambiguous base positions and improving the accuracy of AMR and plasmid prediction from Nanopore assemblies. As the technology continues to evolve, Nanopore sequencing has the potential to serve as a valuable alternative or complementary approach to short-read technologies for comprehensive genomic characterization in epidemiological investigations.

## Supplementary material

10.1099/mgen.0.001626 Uncited Supplementary Material 1.

## Abbreviations

AMR: antimicrobial resistance
CDIP: *Corynebacterium diphtheriae*
CT: complex type
HAC: high-accuracy mode
IFIK: institute for infectious diseases
MLST: multi-locus sequence typing
NGS: next-generation sequencing
ONT: Oxford nanopore technologies
SB: strand bias
ST: sequence type
VRE: vancomycin-resistant enterococci
WGS: whole-genome sequencing
