# Supplementary material for: Evaluation of Oxford Nanopore Technologies workflows for genomic epidemiology of outbreak-associated bacterial isolates in the clinical setting
Source: Microb Genom. 2026 Feb 10;12(2):001626. doi: 10.1099/mgen.0.001626 (PMC12890258; doi:10.1099/mgen.0.001626)
Supplement: Uncited Supplementary Material 1. [file mgen-12-01626-s001.pdf]

Supplementary Material

Evaluation of Oxford Nanopore Technologies workflow for genomic epidemiology of outbreak-associated bacterial isolates in the clinical setting

Stefan Neuenschwander[1], Loïc Borcard[1], Sonja Gempeler[1], Miguel Terrazos Miani[1], Carlo Casanova[1], Alban Ramette[1,2*]

Affiliations
[1] Institute for Infectious Diseases, University of Bern, Bern, Switzerland
[2] Multidisciplinary Center for Infectious Diseases, University of Bern, Bern, Switzerland

*Correspondence: Alban Ramette. Institute for Infectious Diseases, University of Bern, Friedbühlstrasse 25, Bern 3001, Switzerland. Tel +41 31 684 1908, Email: alban.ramette@unibe.ch

Supplementary Figures

A) Percent good cgMLST targets

	VRE_16	VRE_14	VRE_12	VRE_21	VRE_02	VRE_04	VRE_05	VRE_18	VRE_07	VRE_08	VRE_11	VRE_10	VRE_17	VRE_19	VRE_20	VRE_03	VRE_09	VRE_01	VRE_15	VRE_06	count	mean	std	min	25%	50%	75%	max
HAC4k	98.7	99.4	99.2	99.0	99.1	99.2	98.7	97.9	99.2	99.0	99.3	99.3	97.7	94.7	96.3	-	-	98.3	98.2	98.3	18	98.4	1.2	94.7	98.3	98.8	99.2	99.4
SUP4k	98.7	99.4	99.2	99.0	99.2	99.2	98.7	98.0	99.1	99.2	99.3	99.2	97.7	94.9	97.5	-	-	98.5	98.7	98.6	18	98.5	1.0	94.9	98.5	98.8	99.2	99.4
HAC5k	98.6	99.4	99.2	99.0	99.2	99.2	98.5	98.0	99.2	99.1	99.2	99.2	97.7	94.9	99.0	98.2	98.4	98.1	98.7	98.7	20	98.6	1.0	94.9	98.3	98.8	99.2	99.4
SUPD5k	98.7	99.3	99.2	98.9	99.2	99.2	98.6	98.0	99.1	99.2	99.2	99.2	97.5	94.9	99.0	98.2	98.4	98.2	98.5	98.9	20	98.6	1.0	94.9	98.3	98.9	99.2	99.3
SUPD.mod	98.7	99.3	99.2	98.9	99.2	99.2	98.6	97.9	99.1	99.2	99.2	99.3	97.7	94.8	99.0	98.2	98.3	98.2	98.5	98.8	20	98.6	1.0	94.8	98.3	98.8	99.2	99.3
SUPR	98.6	99.4	99.2	99.0	99.2	99.1	98.8	97.8	99.2	99.2	99.0	99.3	97.9	94.9	99.0	98.7	98.6	98.7	98.8	99.2	20	98.7	1.0	94.9	98.6	99.0	99.2	99.4
HAC43	98.6	99.2	99.2	99.0	99.0	99.2	98.7	98.0	99.2	99.2	99.3	99.2	97.4	94.7	99.0	98.3	98.5	98.5	98.8	98.9	20	98.6	1.0	94.7	98.5	98.9	99.2	99.3
SUPD43	98.7	99.3	99.2	98.9	99.2	99.2	98.8	97.9	99.0	99.2	99.1	99.3	97.9	94.9	99.0	98.5	98.7	98.8	99.0	99.2	20	98.7	1.0	94.9	98.7	99.0	99.2	99.3
HAC50	98.6	99.2	99.2	99.0	99.2	99.2	98.7	97.9	99.2	99.0	99.2	99.2	97.7	94.8	98.9	98.6	98.5	98.4	98.6	98.8	20	98.6	1.0	94.8	98.6	98.8	99.2	99.2
SUPD50	98.6	99.4	99.2	99.0	99.2	99.2	98.8	97.9	99.2	99.2	99.2	99.3	97.9	94.9	99.0	98.8	98.5	98.8	99.0	99.3	20	98.7	1.0	94.9	98.8	99.0	99.2	99.4
HAC43.masked	98.2	99.0	98.8	98.7	98.9	98.9	98.0	97.5	98.1	98.5	99.0	98.7	95.6	93.6	98.5	95.6	95.2	96.1	96.1	96.2	20	97.5	1.6	93.6	96.1	98.2	98.7	99.0
SUPD43.masked	98.7	99.3	99.1	99.0	99.2	99.0	98.0	97.7	98.2	98.5	98.7	98.2	96.9	93.8	98.9	96.8	96.6	96.8	97.4	97.3	20	97.9	1.3	93.8	97.2	98.2	98.9	99.3
HACP43	98.6	99.4	99.1	98.9	99.1	99.2	98.7	97.8	99.2	99.2	99.2	99.2	97.8	94.8	99.0	98.8	98.8	98.8	99.0	99.2	20	98.7	1.0	94.8	98.8	99.0	99.2	99.4
SUPDP43	98.7	99.4	99.2	99.0	99.1	99.2	98.8	97.9	99.2	99.2	99.2	99.3	97.9	94.9	99.0	98.9	98.9	98.9	99.0	99.4	20	98.7	1.0	94.9	98.9	99.0	99.2	99.4
SUPD&P43	98.7	99.4	99.2	98.8	99.2	99.2	98.8	97.8	99.2	99.2	99.3	99.3	98.0	94.9	99.0	98.9	98.8	98.7	99.0	99.4	20	98.7	1.0	94.9	98.8	99.0	99.2	99.4
HAC43CC	98.6	99.2	99.2	99.0	99.0	99.2	98.7	97.9	99.1	99.2	99.3	99.2	97.5	94.7	99.0	98.4	98.6	98.5	98.8	99.0	20	98.6	1.0	94.7	98.6	99.0	99.2	99.3
SUPD43CC	98.7	99.3	99.2	98.9	99.2	99.2	98.8	97.8	99.0	99.2	99.1	99.3	97.9	94.9	99.0	98.5	98.7	98.8	99.0	99.2	20	98.7	1.0	94.9	98.7	99.0	99.2	99.3
ILLUM	98.7	99.4	99.2	99.0	99.2	99.2	98.8	98.3	99.2	99.2	99.3	99.3	98.0	94.9	99.0	98.9	98.9	98.9	99.0	99.4	20	98.8	1.0	94.9	98.9	99.0	99.2	99.4

B) Number of alleles different from Illumina

Treatment	VRE_16	VRE_14	VRE_12	VRE_21	VRE_02	VRE_04	VRE_05	VRE_18	VRE_07	VRE_08	VRE_11	VRE_10	VRE_17	VRE_19	VRE_20	VRE_03	VRE_09	VRE_01	VRE_15	VRE_06	count	mean	std	min	25%	50%	75%	max
HAC4k	0	0	0	0	1	0	0	1	4	4	5	4	6	6	19	-	-	27	20	32	18	7.2	9.8	0.0	0.0	4.0	6.0	32.0
SUP4k	0	0	0	0	0	0	2	1	2	2	1	4	6	11	16	-	-	44	39	39	18	9.3	14.7	0.0	0.0	2.0	9.8	44.0
HAC5k	0	0	0	0	0	1	1	1	6	5	9	7	4	6	0	11	16	8	15	14	20	5.2	5.3	0.0	0.0	4.5	8.3	16.0
SUPD5k	0	0	0	0	0	1	0	1	1	2	3	5	16	10	4	30	29	24	28	38	20	9.6	12.5	0.0	0.0	2.5	18.0	38.0
SUPD.mod	0	0	0	0	0	1	1	1	4	5	7	6	4	6	1	12	16	11	17	14	20	5.3	5.6	0.0	0.8	4.0	8.0	17.0
SUPR	0	0	0	0	0	0	1	1	0	0	0	0	5	3	0	10	13	14	13	16	20	3.8	5.6	0.0	0.0	0.0	6.3	16.0
HAC43	0	0	0	0	0	0	0	1	1	0	0	0	11	9	3	22	30	19	19	27	20	7.1	10.1	0.0	0.0	0.5	13.0	30.0
SUPD43	0	0	0	0	0	0	0	1	0	0	0	0	2	1	0	0	6	4	6	3	20	1.2	2.0	0.0	0.0	0.0	1.3	6.0
HAC50	0	0	0	0	0	0	0	1	0	0	0	0	5	6	1	20	16	15	18	20	20	5.1	7.6	0.0	0.0	0.0	8.3	20.0
SUPD50	0	0	0	0	0	0	1	2	0	1	0	0	0	0	0	0	4	1	0	1	20	0.5	1.0	0.0	0.0	0.0	1.0	4.0
HAC43.masked	0	0	0	0	0	0	0	1	0	0	0	0	0	1	0	2	4	3	2	3	20	0.8	1.2	0.0	0.0	0.0	1.3	4.0
SUPD43.masked	0	0	0	0	0	0	0	1	0	0	0	0	0	0	0	0	0	0	0	0	20	0.1	0.2	0.0	0.0	0.0	0.0	1.0
HACP43	0	0	0	0	0	0	0	1	0	0	0	0	0	0	0	0	0	0	0	0	20	0.1	0.2	0.0	0.0	0.0	0.0	1.0
SUPDP43	0	0	0	0	0	0	0	2	0	0	0	0	0	0	0	0	0	0	0	0	20	0.1	0.4	0.0	0.0	0.0	0.0	2.0
SUPD&P43	0	0	0	0	0	0	0	2	0	0	0	0	0	0	0	0	0	0	0	0	20	0.1	0.4	0.0	0.0	0.0	0.0	2.0
HAC43CC	0	0	0	0	0	0	0	1	0	0	0	0	0	0	0	0	0	0	0	0	20	0.1	0.2	0.0	0.0	0.0	0.0	1.0
SUPD43CC	0	0	0	0	0	0	0	1	0	0	0	0	0	0	0	0	0	0	0	0	20	0.1	0.2	0.0	0.0	0.0	0.0	1.0

C) MLST sequence type

Treatment	VRE_16	VRE_14	VRE_12	VRE_21	VRE_02	VRE_04	VRE_05	VRE_18	VRE_07	VRE_08	VRE_11	VRE_10	VRE_17	VRE_19	VRE_20	VRE_03	VRE_09	VRE_01	VRE_15	VRE_06
HAC4k	117	117	117	117	78	117	796	80	117	117	117	117	133	296	117	-	-	796	796	117
SUP4k	117	117	117	117	78	117	796	80	117	117	117	117	133	296	117	-	-	796	796	117
HAC5k	117	117	117	117	78	117	796	80	117	117	117	117	133	296	117	796	796	796	796	117
SUPD5k	117	117	117	117	78	117	796	80	117	117	117	117	133	296	117	796	796	796	796	117
SUPD.mod	117	117	117	117	78	117	796	80	117	117	117	117	133	296	117	796	796	796	796	117
SUPR	117	117	117	117	78	117	796	80	117	117	117	117	133	296	117	796	796	796	796	117
HAC43	117	117	117	117	78	117	796	80	117	117	117	117	133	296	117	796	796	796	796	117
SUPD43	117	117	117	117	78	117	796	80	117	117	117	117	133	296	117	796	796	796	796	117
HAC50	117	117	117	117	78	117	796	80	117	117	117	117	133	296	117	796	796	796	796	117
SUPD50	117	117	117	117	78	117	796	80	117	117	117	117	133	296	117	796	796	796	796	117
HAC43.masked	117	117	117	117	78	117	796	80	117	f	117	117	133	296	117	796	f	796	796	117
SUPD43.masked	117	117	117	117	78	117	796	80	117	117	117	117	133	296	117	796	796	796	796	117
HACP43	117	117	117	117	78	117	796	80	117	117	117	117	133	296	117	796	796	796	796	117
SUPDP43	117	117	117	117	78	117	796	80	117	117	117	117	133	296	117	796	796	796	796	117
SUPD&P43	117	117	117	117	78	117	796	80	117	117	117	117	133	296	117	796	796	796	796	117
SUPD43C	117	117	117	117	78	117	796	80	117	117	117	117	133	296	117	796	796	796	796	117
SUPD43CC	117	117	117	117	78	117	796	80	117	117	117	117	133	296	117	796	796	796	796	117
ILLUM	117	117	117	117	78	117	796	80	117	117	117	117	133	296	117	796	796	796	796	117

D) cgMLST complex type

Treatment	VRE_16	VRE_14	VRE_12	VRE_21	VRE_02	VRE_04	VRE_05	VRE_18	VRE_07	VRE_08	VRE_11	VRE_10	VRE_17	VRE_19	VRE_20	VRE_03	VRE_09	VRE_01	VRE_15	VRE_06
HAC4k	2094	u	1177	5602	1555	1177	1219	1552	4425	4424	1477	u	2888	426	u	-	-	u	u	u
SUP4k	2094	u	1177	5602	1555	1177	1219	1552	4425	4424	1477	u	2888	426	u	-	-	u	u	u
HAC5k	2094	u	1177	5602	1555	1177	1219	1552	4425	4424	1477	u	2888	426	2505	2025	u	2025	u	u
SUPD5k	2094	u	1177	5602	1555	1177	1219	1552	4425	4424	1477	u	2888	426	u		u	u	u	u
SUPD.mod	2094	u	1177	5602	1555	1177	1219	1552	4425	4424	1477	u	2888	426	u	2025	u	2025	u	u
SUPR	2094	u	1177	5602	1555	1177	1219	1552	4425	4424	1477	u	2888	426	2505	2025	2025	2025	u	u
HAC43	2094	u	1177	5602	1555	1177	1219	1552	4425	4424	1477	u	2888	426	7829	u	u	u	u	u
SUPD43	2094	u	1177	5602	1555	1177	1219	1552	4425	4424	1477	u	2888	426	2505	1219	2025	1219	u	u
HAC50	2094	u	1177	5602	1555	1177	1219	1552	4425	4424	1477	u	2888	426	7829	u	u	2025	u	u
SUPD50	2094	u	1177	5602	1555	1177	1219	2887	4425	4424	1477	u	564	426	2505	1219	1219	1219	u	u
HAC43.masked	2094	u	1177	5602	1555	1177	1219	1552	4425	4424	1477	24	564	426	2505	1219	1219	1219	u	u
SUPD43.masked	2094	u	1177	5602	1555	1177	1219	1552	4425	4424	1477	24	564	426	2505	1219	1219	1219	u	u
HACP43	2094	u	1177	5602	1555	1177	1219	1552	4425	4424	1477	u	564	426	2505	1219	1219	1219	u	u
SUPDP43	2094	u	1177	5602	1555	1177	1219	2887	4425	4424	1477	u	564	426	2505	1219	1219	1219	u	u
SUPD&P43	2094	u	1177	5602	1555	1177	1219	2887	4425	4424	1477	u	564	426	2505	1219	1219	1219	u	u
HAC43CC	2094	u	1177	5602	1555	1177	1219	1552	4425	4424	1477	u	564	426	2505	1219	1219	1219	u	u
SUPD43CC	2094	u	1177	5602	1555	1177	1219	1552	4425	4424	1477	u	564	426	2505	1219	1219	1219	u	u
ILLUM	2094	u	1177	5602	1555	1177	1219	2887	4425	4424	1477	u	564	426	2505	1219	1219	1219	u	u

Figure S1. Comparison of cgMLST results for clinical VRE isolates sequenced with Illumina and Oxford Nanopore (ONT). A) Percentage the 1,423 cgMLST target genes that were detected (i.e. passed the QC) in the different assemblies. B) Numbers of mismatching alleles between the ONT and the corresponding Illumina assemblies. C) Detected MLST Sequence types (ST). The letters 'f' indicates failed analysis due to missing target genes. D) Complex types (cgMLST). The letter 'u' indicates unknown CT. Detailed information on the treatments is provided in Table S1.

A: Percent good cgMLST targets

Treatment	CDIP_01	CDIP_02	CDIP_03	CDIP_04	CDIP_05	CDIP_06	CDIP_07	CDIP_08	CDIP_09	CDIP_10	CDIP_11	CDIP_12	CDIP_13	CDIP_14	CDIP_15	CDIP_16	CDIP_17	CDIP_18	CDIP_19	CDIP_20	CDIP_21	count	mean	std	min	25%	50%	75%	max
HAC43	96.2	95.7	95.3	94.9	95.2	96.2	93.9	95.3	96.4	93.8	92.9	95.6	88.2	95.7	89.2	89.3	95.3	95.4	95.9	95.4	89.5	21	94.0	2.6	88.2	93.8	95.3	95.7	96.4
HAC43.masked	95.6	94.9	94.9	94.2	94.8	95.6	93.6	95.0	95.1	93.3	90.6	95.3	86.1	95.4	88.0	88.4	94.9	94.7	93.1	94.4	88.7	21	93.2	2.9	86.1	93.1	94.7	95.0	95.6
HAC4k	95.2	94.7	95.43	95.2	95.5	96.19	89.8	95.43	96.0	91.5	91.1	95.96	88.3	95.88	87.5	87.0	95.0	95.5	93.8	95.5	88.0	21	93.3	3.3	87.0	91.1	95.2	95.5	96.2
HACP43	96.1	96.2	95.27	96.3	95.2	96.19	95.4	95.27	95.7	94.6	95.1	95.66	88.2	95.35	89.3	89.7	95.2	94.4	96.0	95.1	89.6	21	94.3	2.6	88.2	94.6	95.3	95.7	96.3
SUP4k	95.8	95.6	95.73	95.9	95.58	96.27	92.8	95.66	96.3	93.5	93.2	95.96	88.5	96.11	89.0	88.6	95.7	95.7	95.0	95.6	89.0	21	94.1	2.8	88.5	93.2	95.6	95.8	96.3
SUPD43	96.1	96.6	95.27	96.3	95.2	96.19	95.3	95.27	96.6	95.4	94.7	95.58	88.6	95.58	89.8	90.2	95.7	95.7	96.3	95.6	89.8	21	94.6	2.5	88.6	95.2	95.6	96.1	96.6
SUPD43.masked	95.5	95.6	94.9	95.2	94.8	95.7	94.2	95.0	95.9	94.1	93.1	95.3	87.0	95.4	88.4	88.6	95.0	94.9	91.4	94.8	88.7	21	93.5	2.8	87.0	93.1	94.9	95.3	95.9
SUPDP43	96.2	96.2	95.2	96.3	95.2	96.2	95.4	95.3	96.2	95.2	95.4	95.7	88.2	95.4	89.9	89.8	95.2	94.9	96.0	95.1	89.6	21	94.4	2.6	88.2	95.1	95.3	96.0	96.3
HAC50	96.2	95.7	95.3	94.8	95.2	96.2	93.5	95.3	96.2	93.8	92.3	95.7	87.8	95.7	88.6	89.3	95.1	94.9	95.7	95.0	88.9	21	93.8	2.8	87.8	93.5	95.1	95.7	96.2
SUPD50	96.2	96.1	95.3	96.3	95.2	96.2	95.3	95.3	96.2	95.3	94.8	95.7	88.2	95.7	89.6	89.8	95.2	95.2	95.9	95.1	89.6	21	94.4	2.6	88.2	95.1	95.3	95.9	96.3
ILLUM	96.7	96.7	95.8	96.8	95.7	96.7	95.9	95.8	96.7	95.9	95.8	96.2	88.7	96.2	90.4	90.3	95.7	95.7	96.5	95.7	90.1	21	95.0	2.6	88.7	95.7	95.8	96.5	96.8

B: Number of alleles different from Illumina

Treatment	CDIP_01	CDIP_02	CDIP_03	CDIP_04	CDIP_05	CDIP_06	CDIP_07	CDIP_08	CDIP_09	CDIP_10	CDIP_11	CDIP_12	CDIP_13	CDIP_14	CDIP_15	CDIP_16	CDIP_17	CDIP_18	CDIP_19	CDIP_20	CDIP_21	count	mean	std	min	0.25	0.5	0.75	max
HAC43	0	0	0	0	0	0	0	0	0	0	0	0	0	0	0	0	0	0	0	0	0	21	0	0	0	0	0	0	0
HAC43.masked	0	0	0	0	0	0	0	0	0	0	0	0	0	0	0	0	0	0	0	0	0	21	0	0	0	0	0	0	0
HAC4k	0	0	0	0	0	0	0	0	0	1	0	0	0	0	0	0	0	0	0	0	0	21	0.05	0.2	0	0	0	0	1
HACP43	0	0	0	0	0	0	0	0	0	0	0	0	0	0	0	0	0	0	0	0	0	21	0	0	0	0	0	0	0
SUP4k	0	0	0	0	0	0	1	0	0	0	0	0	0	0	0	0	0	0	0	0	0	21	0.05	0.2	0	0	0	0	1
SUPD43	0	0	0	0	0	0	0	0	0	0	0	0	0	0	0	0	0	0	0	0	0	21	0	0	0	0	0	0	0
SUPD43.masked	0	0	0	0	0	0	0	0	0	0	0	0	0	0	0	0	0	0	0	0	0	21	0	0	0	0	0	0	0
SUPDP43	0	0	0	0	0	0	0	0	0	0	0	0	0	0	0	0	0	0	0	0	0	21	0	0	0	0	0	0	0
HAC50	0	0	0	0	0	0	0	0	0	0	0	0	0	0	0	0	0	0	0	0	0	21	0	0	0	0	0	0	0
SUPD50	0	0	0	0	0	0	0	0	0	0	0	0	0	0	0	0	0	0	0	0	0	21	0	0	0	0	0	0	0

C: MLST sequence type

Treatment	CDIP_01	CDIP_02	CDIP_03	CDIP_04	CDIP_05	CDIP_06	CDIP_07	CDIP_08	CDIP_09	CDIP_10	CDIP_11	CDIP_12	CDIP_13	CDIP_14	CDIP_15	CDIP_16	CDIP_17	CDIP_18	CDIP_19	CDIP_20	CDIP_21
HAC43	384	384	377	384	377	384	698	377	384	698	698	377	u	377	574	574	377	377	103	377	574
HAC43.masked	384	384	377	384	377	384	698	377	384	698	698	377	u	377	574	574	377	377	103	377	574
HAC4k	384	384	377	384	377	384	698	377	384	698	698	377	u	377	574	574	377	377	103	377	574
HACP43	384	384	377	384	377	384	698	377	384	698	698	377	u	377	574	574	377	377	103	377	574
SUP4k	384	384	377	384	377	384	698	377	384	698	698	377	u	377	574	574	377	377	103	377	574
SUPD43	384	384	377	384	377	384	698	377	384	698	698	377	u	377	574	574	377	377	103	377	574
SUPD43.masked	384	384	377	384	377	384	698	377	384	698	698	377	u	377	574	574	377	377	103	377	574
SUPDP43	384	384	377	384	377	384	698	377	384	698	698	377	u	377	574	574	377	377	103	377	574
HAC50	384	384	377	384	377	384	698	377	384	698	698	377	u	377	574	574	377	377	103	377	574
SUPD50	384	384	377	384	377	384	698	377	384	698	698	377	u	377	574	574	377	377	103	377	574
ILLUM	384	384	377	384	377	384	698	377	384	698	698	377	u	377	574	574	377	377	103	377	574

Figure S2. Comparison of cgMLST results from 21 clinical *Corynebacterium diphtheriae* isolates sequenced in parallel with Illumina and ONT. A) Percentage the 1,312 cgMLST target genes that were detected (i.e. passed the QC) in the different assemblies. B) Numbers of mismatching alleles between the ONT and the corresponding Illumina assemblies. C) Detected MLST Sequence types (ST). The letters 'u' indicates unknown CT. Detailed information on the treatments is provided in Table S1.

A)

	CDIP_03	CDIP_05	CDIP_07	CDIP_08	CDIP_10	CDIP_11	CDIP_12	CDIP_13	CDIP_14	CDIP_15	CDIP_16	CDIP_17	CDIP_18	CDIP_19	CDIP_20	CDIP_21
AMINOGLYCOSIDE	111	111	111	111	111	111	111	111	111	111	111	111	111		111	111
BETA-LACTAM							111		111							
LINCOSAMIDE/MACROLIDE/STREPTOGRAMIN							111		111					111		
PHENICOL	111	111	111	111	111	111	111	111	111	111	111	111	111	111	111	111
QUATERNARY AMMONIUM	111	111		111			111		111			111	111		111	
SULFONAMIDE	111	111	111	111	111	111	111	111	111	111	111	111	111		111	111
TETRACYCLINE	111	111		111			111		111			111	111	111	111	
TRIMETHOPRIM	111	111		111			111		111			111	111		111	

B)

	CDIP_03	CDIP_05	CDIP_07	CDIP_08	CDIP_10	CDIP_11	CDIP_12	CDIP_13	CDIP_14	CDIP_15	CDIP_16	CDIP_17	CDIP_18	CDIP_19	CDIP_20	CDIP_21
AZITHROMYCIN/CLINDAMYCIN/ERYTHROMYCIN/LINCOMYCIN/STREPTOGRAMIN B/TYLOSIN							111		111					111		
BETA-LACTAM							111		111							
CHLORAMPHENICOL	111	111	111	111	111	111	111	111	111	111	111	111	111	111	111	111
GENTAMICIN/KANAMYCIN/TOBRAMYCIN							111		111							
KANAMYCIN	111	011	111	111	111	111		111		111	111	111	011		111	111
QUATERNARY AMMONIUM	111	111		111			111		111			111	111		111	
STREPTOMYCIN	111	111	111	111	111	111	111	111	111	111	111	111	111		111	111
SULFONAMIDE	111	111	111	111	111	111	111	111	111	111	111	111	111		111	111
TETRACYCLINE	111	111		111			111		111			111	111	111	111	
TRIMETHOPRIM	111	111		111			111		111			111	111		111	

C)

	CDIP_03	CDIP_05	CDIP_07	CDIP_08	CDIP_10	CDIP_11	CDIP_12	CDIP_13	CDIP_14	CDIP_15	CDIP_16	CDIP_17	CDIP_18	CDIP_19	CDIP_20	CDIP_21
aadA1							111		111							
aadA5	111	111		111			111		111			111	111		111	
ant(2'')-Ia							111		111							
aph(3'')-Ib	111	111	111	111	111	111		111		111	111	111	111		111	111
aph(3')-Ia	111	011	111	111	111	111		111		111	111	111	011		111	111
aph(6)-Id	111	111	111	111	111	111		111		111	111	111	111		111	111
blaOXA-2							111		111							
cml	111	111		111			111		111			111	111		111	
cmx	111	111	111	111	111	111		111		111	111	111	111	111	111	111
dfrA1	111	111		111			111		111			111	111		111	
erm(X)							111		111					111		
qacEdelta1	111	111		111			111		111			111	111		111	
sul1	111	111	111	111	111	111	111	111	111	111	111	111	111		111	111
tet(33)	111	111		111			111		111			111	111		111	
tet(O)														111		
tet(W)							111		111							

Figure S3. Comparison of the resistance analysis results of ONT and Illumina based assemblies. Tuples indicate presence (1) or absence (0) in the treatments SUBDP_43 (RPB), SUPD_43 (RBK), and ILLUM (Illumina). A) Antibiotic classes, B) Antibiotic subclasses, C) Resistance genes.

A)

	Chromosome Circular	Chromosome Noncircular	Plasmid Circular	Plasmid Noncircular	AMR Plasmid Noncircular	AMR Plasmid Circular
VRE_01-N	1	0	6	0	0	2
VRE_01-P	0	8	4	4	1	1
VRE_01-X	1	1	6	0	0	2
VRE_02-N	0	6	1	6	2	0
VRE_02-P	0	16	3	6	2	0
VRE_02-X	0	6	4	3	0	2
VRE_03-N	1	0	5	2	0	2
VRE_03-P	0	10	4	6	1	1
VRE_03-X	1	0	4	6	1	1
VRE_04-N	1	0	5	2	0	1
VRE_04-P	0	5	5	3	0	1
VRE_04-X	1	0	6	2	0	1
VRE_05-N	1	0	5	5	1	1
VRE_05-P	0	12	3	7	2	0
VRE_05-X	1	0	4	4	1	1
VRE_06-N	1	2	3	4	0	1
VRE_06-P	0	11	3	6	1	0
VRE_06-X	1	2	3	3	0	1
VRE_07-N	0	6	2	4	0	1
VRE_07-P	0	15	2	9	0	1
VRE_07-X	0	5	2	5	0	1

	Chromosome Circular	Chromosome Noncircular	Plasmid Circular	Plasmid Noncircular	AMR Plasmid Noncircular	AMR Plasmid Circular
VRE_16-N	1	0	5	0	0	1
VRE_16-P	0	6	3	2	1	0
VRE_16-X	1	0	6	0	0	1
VRE_17-N	1	0	1	0	0	1
VRE_17-P	0	6	1	0	0	1
VRE_17-X	1	0	1	0	0	1
VRE_18-N	1	0	6	3	1	1
VRE_18-P	0	10	4	6	1	1
VRE_18-X	1	1	6	4	1	1
VRE_19-N	1	0	1	0	0	0
VRE_19-P	0	2	1	0	0	0
VRE_19-X	1	0	1	0	0	0
VRE_20-N	0	2	5	1	0	2
VRE_20-P	0	11	3	11	2	0
VRE_20-X	0	2	5	4	0	2
VRE_21-N	1	4	4	2	0	2
VRE_21-P	0	9	7	2	0	2
VRE_21-X	1	2	5	4	0	2

	Chromosome Circular	Chromosome Noncircular	Plasmid Circular	Plasmid Noncircular	AMR Plasmid Noncircular	AMR Plasmid Circular
VRE_08-N	1	0	8	2	0	2
VRE_08-P	0	10	5	5	1	1
VRE_08-X	1	0	8	3	0	2
VRE_09-N	1	0	6	1	0	2
VRE_09-P	0	9	5	4	1	1
VRE_09-X	1	0	7	1	0	2
VRE_10-N	0	3	1	3	0	1
VRE_10-P	0	17	1	4	0	1
VRE_10-X	0	4	1	5	1	1
VRE_11-N	1	0	6	1	0	1
VRE_11-P	0	8	5	2	0	1
VRE_11-X	1	1	4	3	0	1
VRE_12-N	1	1	5	2	0	1
VRE_12-P	0	7	3	4	0	1
VRE_12-X	0	3	6	2	0	1
VRE_14-N	0	3	3	4	1	0
VRE_14-P	0	13	4	3	0	0
VRE_14-X	0	2	3	5	0	1
VRE_15-N	1	1	3	7	2	0
VRE_15-P	0	15	2	5	2	0
VRE_15-X	1	1	3	7	2	0

B)

Number of replicons

[min/max/average]

	RBK	RPB	MIXED
Chromosome circular	0/1/0.75^{a}	0/0/0^{a}	0/1/0.7^{a}
Chromosome noncircular	0/6/1.4^{b}	2/17/10^{a}	0/6/1.5^{b}
Plasmid circular	1/8/4.05^{a}	1/7/3.4^{a}	1/8/4.25^{a}
Plasmid noncircular	0/7/2.45^{b}	0/11/4.45^{a}	0/7/3.05^{ab}
AMR_Plasmid_Circular	0/2/1.1^{ab}	0/2/0.65^{a}	0/2/1.2^{b}
AMR_Plasmid_Noncircular	0/2/0.35^{a}	0/2/0.75^{a}	0/2/0.3^{a}

Figure S4. Assembly performance of PCR-based, PCR-free, and mixed Nanopore reads assembled by the stand-alone assembly pipeline used in this study (see Methods section). Contigs reported as circular by Flye were annotated by appending [topology=circular] to their FASTA headers. The plasmid analysis was conducted in SeqSphere using the task templates 'Chromosome & plasmid typing' version 1.1, 'CGE MobileElementFinder' version 1.0, and 'NCBI AMRFinderPlus' version 1.2. **A**) Numbers of contigs by categories based on replicon type, circularity and presence of AMR genes. **B**) Summary of the data provided in A). Letters "a", "b", "c" denote statistically distinct groups (Mann-Whitney U test; corrected p-value for multiple testing, $p<0.05$). Grey shadings indicate rows with significant differences between treatments.

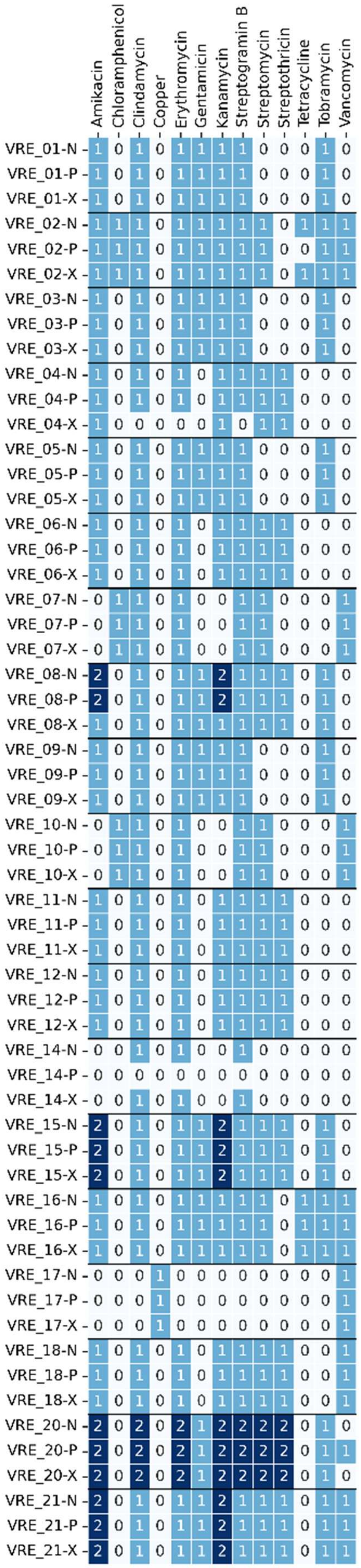

Figure S5. Plasmid-borne resistance based on assemblies produced by the stand-alone assembly pipeline used in this study, followed by SeqSphere analysis (see legend of Figure S4). Numbers represent counts of resistance genes detected for each specific antibiotic subclass.

Supplementary Tables

Table S1. Overview of experimental parameters evaluated in the study.

Samples	Treatment name	Kit	PCR	GridION release	Basecaller	Mode	Model	Sampling rate	Flye parameters	Medaka model
VRE	HAC4k	SQK-RBK114.96	no	22.10.7	Guppy 6.3.9	HAC	default, real-time basecalling	4khz	--nano-raw	r1041_e82_260bps_hac_g632
VRE	SUP4k	SQK-RBK114.96	no	22.10.7	Guppy 6.3.9	SUP	Default	4khz	--nano-hq --read-error 0.03	r1041_e82_260bps_sup_g632
VRE	HAC5k	SQK-RBK114.96	no	23.04.6	Guppy 6.5.7	HAC	default, real-time basecalling	5khz	--nano-raw	r1041_e82_400bps_hac_v4.2.0
VRE	SUPD5k	SQK-RBK114.96	no	23.04.6	Dorado 0.3.3	SUP	dna_r10.4.1_e8.2_400bps_sup@v4.2.0	5khz	--nano-hq --read-error 0.03	r1041_e82_400bps_sup_v4.2.0
VRE	SUPD.mod	SQK-RBK114.96	no	23.04.6	Dorado 0.3.3	SUP modified bases	dna_r10.4.1_e8.2_400bps_sup@v4.2.0 + 5mC +6mA	5khz	--nano-hq --read-error 0.03	r1041_e82_400bps_sup_v4.2.0
VRE	SUPR	SQK-RBK114.96	no	23.04.6	Dorado 0.4.0	SUP	res_dna_r10.4.1_e8.2_400bps_sup@2023-09-22_bacterial-methylation	5khz	--nano-hq --read-error 0.03	r1041_e82_400bps_sup_v4.2.0
VRE	HAC43	SQK-RBK114.96	no	23.04.6	Dorado 0.5.0	HAC	dna_r10.4.1_e8.2_400bps_hac@v4.3.0	5khz	--nano-raw	r1041_e82_400bps_hac_v4.3.0
VRE	SUPD43	SQK-RBK114.96	no	23.04.6	Dorado 0.5.0	SUP	dna_r10.4.1_e8.2_400bps_sup@v4.3.0	5khz	--nano-hq --read-error 0.03	r1041_e82_400bps_sup_v4.3.0
VRE	HACP43	SQK-RPB114.24	yes	23.11.7	Dorado 0.5.0	HAC	dna_r10.4.1_e8.2_400bps_hac@v4.3.0	5khz	--nano-raw	r1041_e82_400bps_hac_v4.3.0
VRE	SUPDP43	SQK-RPB114.24	yes	23.11.7	Dorado 0.5.0	SUP	dna_r10.4.1_e8.2_400bps_sup@v4.3.0	5khz	--nano-hq --read-error 0.03	r1041_e82_400bps_sup_v4.3.0
VRE	SUPD&P43	70% SQK-RBK114.96, 30% SQK-RPB114.24	Previously produced sequencing reads were pooled before assembly. See SUPD43 and SUPDP43 above.							
VRE	HAC43CC	Assembly: SQK-RBK114.96, Correction: SQK-RPB114.24	Previously produced assemblies were corrected with previously produced read sets. See HAC43 and HAC43 above.							
VRE	SUPD43CC	Assembly: SQK-RBK114.96, Correction: SQK-RPB114.24	Previously produced assemblies were corrected with previously produced read sets. See SUPD43 and SUPDP43 above.							
VRE	SUPD50	SQK-RBK114.96	no	23.04.6	Dorado 0.7.0	SUP	dna_r10.4.1_e8.2_400bps_sup@v5.0.0	5khz	--nano-hq --read-error 0.03	r1041_e82_400bps_sup_v5.0.0'
VRE	HAC50	SQK-RBK114.96	no	23.04.6	Dorado 0.7.0	HAC	dna_r10.4.1_e8.2_400bps_hac@v5.0.0	5khz	--nano-raw	r1041_e82_400bps_hac_v5.0.0'
VRE	ILLUM	Illumina DNA	no	-	-	2 x 150	-	-		
CDIP	HAC4k	SQK-RBK114.96	no	22.12.5	Guppy 6.4.6-1	HAC	default, real-time basecalling	4khz	--nano-raw	r1041_e82_260bps_hac_g632
CDIP	SUP4k	SQK-RBK114.96	no	22.12.5	Guppy 6.4.6-1	SUP	default	4khz	--nano-hq --read-error 0.03	r1041_e82_260bps_sup_g632
CDIP	HAC43	SQK-RBK114.96	no	23.07.12, 23.07.12	Dorado 0.5.0	HAC	dna_r10.4.1_e8.2_400bps_hac@v4.3.0	5khz	--nano-raw	r1041_e82_400bps_hac_v4.3.0
CDIP	SUPD43	SQK-RBK114.96	no	23.07.12, 23.07.12	Dorado 0.5.0	SUP	dna_r10.4.1_e8.2_400bps_sup@v4.3.0	5khz	--nano-hq --read-error 0.03	r1041_e82_400bps_sup_v4.3.0
CDIP	ILLUM	Illumina DNA	no	-	-	2 x 150	-	-		

Abbreviations: VRE: Vancomycin resistant *Enterococcus*; CDIP: *Corynebacterium diphtheriae*; Treatment name: A string representing a specific combination of analysis parameters; HAC: High accuracy basecalling mode; SUP: Super high accuracy basecalling mode.

Table S2. Sequencing reads deposited at NCBI short read archive (SRA). Additional datasets are available upon request.

Sample	Treatment	BioProject accession	BioSample accession	SRA accession	SRA study	SRA library ID
VRE_01	SUPD43	PRJNA1230056	SAMN48144070	SRR36284466	SRP649163	VRE_01_SUPD43_IFIK
VRE_02	SUPD43	PRJNA1230056	SAMN48144071	SRR36284465	SRP649163	VRE_02_SUPD43_IFIK
VRE_03	SUPD43	PRJNA1230056	SAMN48144072	SRR36284454	SRP649163	VRE_03_SUPD43_IFIK
VRE_04	SUPD43	PRJNA1230056	SAMN48144073	SRR36284453	SRP649163	VRE_04_SUPD43_IFIK
VRE_05	SUPD43	PRJNA1230056	SAMN48144074	SRR36284452	SRP649163	VRE_05_SUPD43_IFIK
VRE_06	SUPD43	PRJNA1230056	SAMN48144075	SRR36284451	SRP649163	VRE_06_SUPD43_IFIK
VRE_07	SUPD43	PRJNA1230056	SAMN48144076	SRR36284450	SRP649163	VRE_07_SUPD43_IFIK
VRE_08	SUPD43	PRJNA1230056	SAMN48144077	SRR36284449	SRP649163	VRE_08_SUPD43_IFIK
VRE_09	SUPD43	PRJNA1230056	SAMN48144078	SRR36284448	SRP649163	VRE_09_SUPD43_IFIK
VRE_10	SUPD43	PRJNA1230056	SAMN48144079	SRR36284447	SRP649163	VRE_10_SUPD43_IFIK
VRE_11	SUPD43	PRJNA1230056	SAMN48144080	SRR36284464	SRP649163	VRE_11_SUPD43_IFIK
VRE_12	SUPD43	PRJNA1230056	SAMN48144081	SRR36284463	SRP649163	VRE_12_SUPD43_IFIK
VRE_14	SUPD43	PRJNA1230056	SAMN48144082	SRR36284462	SRP649163	VRE_14_SUPD43_IFIK
VRE_15	SUPD43	PRJNA1230056	SAMN48144083	SRR36284461	SRP649163	VRE_15_SUPD43_IFIK
VRE_16	SUPD43	PRJNA1230056	SAMN48144084	SRR36284460	SRP649163	VRE_16_SUPD43_IFIK
VRE_17	SUPD43	PRJNA1230056	SAMN48144085	SRR36284459	SRP649163	VRE_17_SUPD43_IFIK
VRE_18	SUPD43	PRJNA1230056	SAMN48144086	SRR36284458	SRP649163	VRE_18_SUPD43_IFIK
VRE_19	SUPD43	PRJNA1230056	SAMN48144087	SRR36284457	SRP649163	VRE_19_SUPD43_IFIK
VRE_20	SUPD43	PRJNA1230056	SAMN48144088	SRR36284456	SRP649163	VRE_20_SUPD43_IFIK
VRE_21	SUPD43	PRJNA1230056	SAMN48144089	SRR36284455	SRP649163	VRE_21_SUPD43_IFIK
VRE_01	SUPDP43	PRJNA1230056	SAMN48144070	SRR36286194	SRP649163	VRE_01_SUPDP43_IFIK
VRE_02	SUPDP43	PRJNA1230056	SAMN48144071	SRR36286193	SRP649163	VRE_02_SUPDP43_IFIK
VRE_03	SUPDP43	PRJNA1230056	SAMN48144072	SRR36286182	SRP649163	VRE_03_SUPDP43_IFIK
VRE_04	SUPDP43	PRJNA1230056	SAMN48144073	SRR36286181	SRP649163	VRE_04_SUPDP43_IFIK
VRE_05	SUPDP43	PRJNA1230056	SAMN48144074	SRR36286180	SRP649163	VRE_05_SUPDP43_IFIK
VRE_06	SUPDP43	PRJNA1230056	SAMN48144075	SRR36286179	SRP649163	VRE_06_SUPDP43_IFIK
VRE_07	SUPDP43	PRJNA1230056	SAMN48144076	SRR36286178	SRP649163	VRE_07_SUPDP43_IFIK
VRE_08	SUPDP43	PRJNA1230056	SAMN48144077	SRR36286177	SRP649163	VRE_08_SUPDP43_IFIK
VRE_09	SUPDP43	PRJNA1230056	SAMN48144078	SRR36286176	SRP649163	VRE_09_SUPDP43_IFIK
VRE_10	SUPDP43	PRJNA1230056	SAMN48144079	SRR36286175	SRP649163	VRE_10_SUPDP43_IFIK
VRE_11	SUPDP43	PRJNA1230056	SAMN48144080	SRR36286192	SRP649163	VRE_11_SUPDP43_IFIK
VRE_12	SUPDP43	PRJNA1230056	SAMN48144081	SRR36286191	SRP649163	VRE_12_SUPDP43_IFIK
VRE_14	SUPDP43	PRJNA1230056	SAMN48144082	SRR36286190	SRP649163	VRE_14_SUPDP43_IFIK
VRE_15	SUPDP43	PRJNA1230056	SAMN48144083	SRR36286189	SRP649163	VRE_15_SUPDP43_IFIK
VRE_16	SUPDP43	PRJNA1230056	SAMN48144084	SRR36286188	SRP649163	VRE_16_SUPDP43_IFIK
VRE_17	SUPDP43	PRJNA1230056	SAMN48144085	SRR36286187	SRP649163	VRE_17_SUPDP43_IFIK
VRE_18	SUPDP43	PRJNA1230056	SAMN48144086	SRR36286186	SRP649163	VRE_18_SUPDP43_IFIK
VRE_19	SUPDP43	PRJNA1230056	SAMN48144087	SRR36286185	SRP649163	VRE_19_SUPDP43_IFIK
VRE_20	SUPDP43	PRJNA1230056	SAMN48144088	SRR36286184	SRP649163	VRE_20_SUPDP43_IFIK
VRE_21	SUPDP43	PRJNA1230056	SAMN48144089	SRR36286183	SRP649163	VRE_21_SUPDP43_IFIK
VRE_01	SUPD50	PRJNA1230056	SAMN48144070	SRR36202318	SRP649163	VRE_01_SUPD50_IFIK
VRE_02	SUPD50	PRJNA1230056	SAMN48144071	SRR36202317	SRP649163	VRE_02_SUPD50_IFIK
VRE_03	SUPD50	PRJNA1230056	SAMN48144072	SRR36202306	SRP649163	VRE_03_SUPD50_IFIK
VRE_04	SUPD50	PRJNA1230056	SAMN48144073	SRR36202305	SRP649163	VRE_04_SUPD50_IFIK
VRE_05	SUPD50	PRJNA1230056	SAMN48144074	SRR36202304	SRP649163	VRE_05_SUPD50_IFIK
VRE_06	SUPD50	PRJNA1230056	SAMN48144075	SRR36202303	SRP649163	VRE_06_SUPD50_IFIK
VRE_07	SUPD50	PRJNA1230056	SAMN48144076	SRR36202302	SRP649163	VRE_07_SUPD50_IFIK
VRE_08	SUPD50	PRJNA1230056	SAMN48144077	SRR36202301	SRP649163	VRE_08_SUPD50_IFIK
VRE_09	SUPD50	PRJNA1230056	SAMN48144078	SRR36202300	SRP649163	VRE_09_SUPD50_IFIK
VRE_10	SUPD50	PRJNA1230056	SAMN48144079	SRR36202299	SRP649163	VRE_10_SUPD50_IFIK
VRE_11	SUPD50	PRJNA1230056	SAMN48144080	SRR36202316	SRP649163	VRE_11_SUPD50_IFIK
VRE_12	SUPD50	PRJNA1230056	SAMN48144081	SRR36202315	SRP649163	VRE_12_SUPD50_IFIK
VRE_14	SUPD50	PRJNA1230056	SAMN48144082	SRR36202314	SRP649163	VRE_14_SUPD50_IFIK
VRE_15	SUPD50	PRJNA1230056	SAMN48144083	SRR36202313	SRP649163	VRE_15_SUPD50_IFIK
VRE_16	SUPD50	PRJNA1230056	SAMN48144084	SRR36202312	SRP649163	VRE_16_SUPD50_IFIK
VRE_17	SUPD50	PRJNA1230056	SAMN48144085	SRR36202311	SRP649163	VRE_17_SUPD50_IFIK

VRE_18	SUPD50	PRJNA1230056	SAMN48144086	SRR36202310	SRP649163	VRE_18_SUPD50_IFIK
VRE_19	SUPD50	PRJNA1230056	SAMN48144087	SRR36202309	SRP649163	VRE_19_SUPD50_IFIK
VRE_20	SUPD50	PRJNA1230056	SAMN48144088	SRR36202308	SRP649163	VRE_20_SUPD50_IFIK
VRE_21	SUPD50	PRJNA1230056	SAMN48144089	SRR36202307	SRP649163	VRE_21_SUPD50_IFIK
VRE_01	Illumina	PRJNA1230056	SAMN48144070	SRR36372753	SRP649163	VRE_01_Illumina_IFIK
VRE_02	Illumina	PRJNA1230056	SAMN48144071	SRR36372752	SRP649163	VRE_02_Illumina_IFIK
VRE_03	Illumina	PRJNA1230056	SAMN48144072	SRR36372742	SRP649163	VRE_03_Illumina_IFIK
VRE_04	Illumina	PRJNA1230056	SAMN48144073	SRR36372741	SRP649163	VRE_04_Illumina_IFIK
VRE_05	Illumina	PRJNA1230056	SAMN48144074	SRR36372740	SRP649163	VRE_05_Illumina_IFIK
VRE_06	Illumina	PRJNA1230056	SAMN48144075	SRR36372739	SRP649163	VRE_06_Illumina_IFIK
VRE_07	Illumina	PRJNA1230056	SAMN48144076	SRR36372738	SRP649163	VRE_07_Illumina_IFIK
VRE_08	Illumina	PRJNA1230056	SAMN48144077	SRR36372737	SRP649163	VRE_08_Illumina_IFIK
VRE_09	Illumina	PRJNA1230056	SAMN48144078	SRR36372736	SRP649163	VRE_09_Illumina_IFIK
VRE_10	Illumina	PRJNA1230056	SAMN48144079	SRR36372735	SRP649163	VRE_10_Illumina_IFIK
VRE_11	Illumina	PRJNA1230056	SAMN48144080	SRR36372751	SRP649163	VRE_11_Illumina_IFIK
VRE_12	Illumina	PRJNA1230056	SAMN48144081	SRR36372750	SRP649163	VRE_12_Illumina_IFIK
VRE_14	Illumina	PRJNA1230056	SAMN48144082	SRR36372749	SRP649163	VRE_14_Illumina_IFIK
VRE_15	Illumina	PRJNA1230056	SAMN48144083	SRR36372748	SRP649163	VRE_15_Illumina_IFIK
VRE_16	Illumina	PRJNA1230056	SAMN48144084	SRR36372747	SRP649163	VRE_16_Illumina_IFIK
VRE_17	Illumina	PRJNA1230056	SAMN48144085	SRR36372746	SRP649163	VRE_17_Illumina_IFIK
VRE_18	Illumina	PRJNA1230056	SAMN48144086	SRR36372745	SRP649163	VRE_18_Illumina_IFIK
VRE_19	Illumina	PRJNA1230056	SAMN48144087	SRR36372744	SRP649163	VRE_19_Illumina_IFIK
VRE_20	Illumina	PRJNA1230056	SAMN48144088	SRR36372743	SRP649163	VRE_20_Illumina_IFIK
CDIP_01	SUPD43	PRJNA1230056	SAMN31250445	SRR36403653	SRP649163	CDIP_01_SUPD43
CDIP_02	SUPD43	PRJNA1230056	SAMN31250446	SRR36403652	SRP649163	CDIP_02_SUPD43
CDIP_03	SUPD43	PRJNA1230056	SAMN31250449	SRR36403641	SRP649163	CDIP_03_SUPD43
CDIP_04	SUPD43	PRJNA1230056	SAMN31250448	SRR36403639	SRP649163	CDIP_04_SUPD43
CDIP_05	SUPD43	PRJNA1230056	SAMN31250453	SRR36403638	SRP649163	CDIP_05_SUPD43
CDIP_06	SUPD43	PRJNA1230056	SAMN31250451	SRR36403637	SRP649163	CDIP_06_SUPD43
CDIP_07	SUPD43	PRJNA1230056	SAMN31250450	SRR36403636	SRP649163	CDIP_07_SUPD43
CDIP_08	SUPD43	PRJNA1230056	SAMN31250452	SRR36403635	SRP649163	CDIP_08_SUPD43
CDIP_09	SUPD43	PRJNA1230056	SAMN31250454	SRR36403634	SRP649163	CDIP_09_SUPD43
CDIP_10	SUPD43	PRJNA1230056	SAMN31250455	SRR36403633	SRP649163	CDIP_10_SUPD43
CDIP_11	SUPD43	PRJNA1230056	SAMN31250456	SRR36403651	SRP649163	CDIP_11_SUPD43
CDIP_12	SUPD43	PRJNA1230056	SAMN31250457	SRR36403650	SRP649163	CDIP_12_SUPD43
CDIP_13	SUPD43	PRJNA1230056	SAMN31250459	SRR36403649	SRP649163	CDIP_13_SUPD43
CDIP_14	SUPD43	PRJNA1230056	SAMN31250458	SRR36403648	SRP649163	CDIP_14_SUPD43
CDIP_15	SUPD43	PRJNA1230056	SAMN31250460	SRR36403647	SRP649163	CDIP_15_SUPD43
CDIP_16	SUPD43	PRJNA1230056	SAMN31250461	SRR36403646	SRP649163	CDIP_16_SUPD43
CDIP_17	SUPD43	PRJNA1230056	SAMN31250462	SRR36403645	SRP649163	CDIP_17_SUPD43
CDIP_18	SUPD43	PRJNA1230056	SAMN31250463	SRR36403644	SRP649163	CDIP_18_SUPD43
CDIP_19	SUPD43	PRJNA1230056	SAMN31250464	SRR36403643	SRP649163	CDIP_19_SUPD43
CDIP_20	SUPD43	PRJNA1230056	SAMN31250465	SRR36403642	SRP649163	CDIP_20_SUPD43
CDIP_21	SUPD43	PRJNA1230056	SAMN31250466	SRR36403640	SRP649163	CDIP_21_SUPD43
CDIP_01	SUPDP43	PRJNA1230056	SAMN31250445	SRR36405621	SRP649163	CDIP_01_SUPDP43
CDIP_02	SUPDP43	PRJNA1230056	SAMN31250446	SRR36405620	SRP649163	CDIP_02_SUPDP43
CDIP_03	SUPDP43	PRJNA1230056	SAMN31250449	SRR36405609	SRP649163	CDIP_03_SUPDP43
CDIP_04	SUPDP43	PRJNA1230056	SAMN31250448	SRR36405607	SRP649163	CDIP_04_SUPDP43
CDIP_05	SUPDP43	PRJNA1230056	SAMN31250453	SRR36405606	SRP649163	CDIP_05_SUPDP43
CDIP_06	SUPDP43	PRJNA1230056	SAMN31250451	SRR36405605	SRP649163	CDIP_06_SUPDP43
CDIP_07	SUPDP43	PRJNA1230056	SAMN31250450	SRR36405604	SRP649163	CDIP_07_SUPDP43
CDIP_08	SUPDP43	PRJNA1230056	SAMN31250452	SRR36405603	SRP649163	CDIP_08_SUPDP43
CDIP_09	SUPDP43	PRJNA1230056	SAMN31250454	SRR36405602	SRP649163	CDIP_09_SUPDP43
CDIP_10	SUPDP43	PRJNA1230056	SAMN31250455	SRR36405601	SRP649163	CDIP_10_SUPDP43
CDIP_11	SUPDP43	PRJNA1230056	SAMN31250456	SRR36405619	SRP649163	CDIP_11_SUPDP43
CDIP_12	SUPDP43	PRJNA1230056	SAMN31250457	SRR36405618	SRP649163	CDIP_12_SUPDP43
CDIP_13	SUPDP43	PRJNA1230056	SAMN31250459	SRR36405617	SRP649163	CDIP_13_SUPDP43
CDIP_14	SUPDP43	PRJNA1230056	SAMN31250458	SRR36405616	SRP649163	CDIP_14_SUPDP43
CDIP_15	SUPDP43	PRJNA1230056	SAMN31250460	SRR36405615	SRP649163	CDIP_15_SUPDP43
CDIP_16	SUPDP43	PRJNA1230056	SAMN31250461	SRR36405614	SRP649163	CDIP_16_SUPDP43

CDIP_17	SUPDP43	PRJNA1230056	SAMN31250462	SRR36405613	SRP649163	CDIP_17_SUPDP43
CDIP_18	SUPDP43	PRJNA1230056	SAMN31250463	SRR36405612	SRP649163	CDIP_18_SUPDP43
CDIP_19	SUPDP43	PRJNA1230056	SAMN31250464	SRR36405611	SRP649163	CDIP_19_SUPDP43
CDIP_20	SUPDP43	PRJNA1230056	SAMN31250465	SRR36405610	SRP649163	CDIP_20_SUPDP43
CDIP_21	SUPDP43	PRJNA1230056	SAMN31250466	SRR36405608	SRP649163	CDIP_21_SUPDP43
CDIP_01	SUPD50	PRJNA1230056	SAMN31250445	SRR36405021	SRP649163	CDIP_01_SUPD50
CDIP_02	SUPD50	PRJNA1230056	SAMN31250446	SRR36405020	SRP649163	CDIP_02_SUPD50
CDIP_03	SUPD50	PRJNA1230056	SAMN31250449	SRR36405009	SRP649163	CDIP_03_SUPD50
CDIP_04	SUPD50	PRJNA1230056	SAMN31250448	SRR36405007	SRP649163	CDIP_04_SUPD50
CDIP_05	SUPD50	PRJNA1230056	SAMN31250453	SRR36405006	SRP649163	CDIP_05_SUPD50
CDIP_06	SUPD50	PRJNA1230056	SAMN31250451	SRR36405005	SRP649163	CDIP_06_SUPD50
CDIP_07	SUPD50	PRJNA1230056	SAMN31250450	SRR36405004	SRP649163	CDIP_07_SUPD50
CDIP_08	SUPD50	PRJNA1230056	SAMN31250452	SRR36405003	SRP649163	CDIP_08_SUPD50
CDIP_09	SUPD50	PRJNA1230056	SAMN31250454	SRR36405002	SRP649163	CDIP_09_SUPD50
CDIP_10	SUPD50	PRJNA1230056	SAMN31250455	SRR36405001	SRP649163	CDIP_10_SUPD50
CDIP_11	SUPD50	PRJNA1230056	SAMN31250456	SRR36405019	SRP649163	CDIP_11_SUPD50
CDIP_12	SUPD50	PRJNA1230056	SAMN31250457	SRR36405018	SRP649163	CDIP_12_SUPD50
CDIP_13	SUPD50	PRJNA1230056	SAMN31250459	SRR36405017	SRP649163	CDIP_13_SUPD50
CDIP_14	SUPD50	PRJNA1230056	SAMN31250458	SRR36405016	SRP649163	CDIP_14_SUPD50
CDIP_15	SUPD50	PRJNA1230056	SAMN31250460	SRR36405015	SRP649163	CDIP_15_SUPD50
CDIP_16	SUPD50	PRJNA1230056	SAMN31250461	SRR36405014	SRP649163	CDIP_16_SUPD50
CDIP_17	SUPD50	PRJNA1230056	SAMN31250462	SRR36405013	SRP649163	CDIP_17_SUPD50
CDIP_18	SUPD50	PRJNA1230056	SAMN31250463	SRR36405012	SRP649163	CDIP_18_SUPD50
CDIP_19	SUPD50	PRJNA1230056	SAMN31250464	SRR36405011	SRP649163	CDIP_19_SUPD50
CDIP_20	SUPD50	PRJNA1230056	SAMN31250465	SRR36405010	SRP649163	CDIP_20_SUPD50
CDIP_21	SUPD50	PRJNA1230056	SAMN31250466	SRR36405008	SRP649163	CDIP_21_SUPD50
CDIP_01	ILLUM	PRJNA889706	SAMN31250445	SRR21870465	SRP402096	ETQTR9770Z_17
CDIP_02	ILLUM	PRJNA889706	SAMN31250446	SRR21870464	SRP402096	ETQTR9770Z_86
CDIP_03	ILLUM	PRJNA889706	SAMN31250449	SRR21870472	SRP402096	YHZKU1889A_52
CDIP_04	ILLUM	PRJNA889706	SAMN31250448	SRR21870482	SRP402096	ABUIM4856I_58
CDIP_05	ILLUM	PRJNA889706	SAMN31250453	SRR21870470	SRP402096	ZJBXV8895B_64
CDIP_06	ILLUM	PRJNA889706	SAMN31250451	SRR21870467	SRP402096	DZFPS1693X_65
CDIP_07	ILLUM	PRJNA889706	SAMN31250450	SRR21870462	SRP402096	JCRCV9467B_66
CDIP_08	ILLUM	PRJNA889706	SAMN31250452	SRR21870477	SRP402096	RAFEP1907X_67
CDIP_09	ILLUM	PRJNA889706	SAMN31250454	SRR21870478	SRP402096	QXTNQ3310Y_17
CDIP_10	ILLUM	PRJNA889706	SAMN31250455	SRR21870479	SRP402096	OKCEL6670K_58
CDIP_11	ILLUM	PRJNA889706	SAMN31250456	SRR21870480	SRP402096	OKCEL6670K_52
CDIP_12	ILLUM	PRJNA889706	SAMN31250457	SRR21870474	SRP402096	XOVVI2310N_71
CDIP_13	ILLUM	PRJNA889706	SAMN31250459	SRR21870463	SRP402096	GVUBY4395B_94
CDIP_14	ILLUM	PRJNA889706	SAMN31250458	SRR21870475	SRP402096	XOVVI2310N_70
CDIP_15	ILLUM	PRJNA889706	SAMN31250460	SRR21870468	SRP402096	DZBRU7614B_30
CDIP_16	ILLUM	PRJNA889706	SAMN31250461	SRR21870471	SRP402096	DZBRU7614B_10
CDIP_17	ILLUM	PRJNA889706	SAMN31250462	SRR21870473	SRP402096	YHWXK8946J_70
CDIP_18	ILLUM	PRJNA889706	SAMN31250463	SRR21870466	SRP402096	EDFQY4895Y_95
CDIP_19	ILLUM	PRJNA889706	SAMN31250464	SRR21870481	SRP402096	NDZRZ6050V_23
CDIP_20	ILLUM	PRJNA889706	SAMN31250465	SRR21870476	SRP402096	TVJEP2910E_08
CDIP_21	ILLUM	PRJNA889706	SAMN31250466	SRR21870469	SRP402096	ZRHNH1248J_23
